# Epididymis-specific RNase A family genes regulate fertility and small RNA processing

**DOI:** 10.1016/j.jbc.2024.107933

**Published:** 2024-10-28

**Authors:** Joshua F. Shaffer, Alka Gupta, Geetika Kharkwal, Edgardo E. Linares, Andrew D. Holmes, Julian R. Swartz, Sol Katzman, Upasna Sharma

**Affiliations:** 1 Department of Molecular, Cell and Developmental Biology, University of California, Santa Cruz, California, USA; 2 Boost Neuroscience Inc, Menlo Park, California, USA; 3 University of Colorado Anshutz Medical Campus, Aurora, Colorado, USA; 4 Genomics Institute, University of California, Santa Cruz, California, USA

**Keywords:** fertility, reproduction, small RNAs, ribonucleases, sperm, epididymis

## Abstract

Sperm small RNAs are implicated in intergenerational transmission of paternal environmental effects. Small RNAs generated by the cleavage of tRNAs, known as tRNA fragments (tRFs) or tRNA-derived RNAs (tDRs or tsRNAs), are an abundant class of RNAs in mature sperm and can be modulated by environmental conditions. The biogenesis of tRFs in the male reproductive tract remains poorly understood. Angiogenin, a member of the ribonuclease A superfamily (RNase A), cleaves tRNAs to generate tRFs in response to cellular stress. Four paralogs of Angiogenin, namely *Rnase9*, *Rnase10*, *Rnase11*, and *Rnase12*, are specifically expressed in the epididymis—a long, convoluted tubule where sperm mature and acquire fertility and motility. Here, by generating mice deleted for all four genes (*Rnase9-12*−/−, termed “KO” for Knock Out), we report that these genes regulate fertility and small RNA levels. KO male mice are sterile; KO sperm fertilized oocytes *in vitro* but failed to efficiently fertilize oocytes *in vivo* due to an inability of sperm to pass through the utero-tubular junction. Intriguingly, there were decreased levels of tRFs and rRNAs (rRNA-derived small RNAs or rsRNAs) in the KO epididymis and epididymal luminal fluid, although RNases 9-12 did not show ribonucleolytic activity *in vitro*. Importantly, KO sperm showed a dramatic decrease in the levels of tRFs, demonstrating a role of epididymis-specific *Rnase9-12* genes in regulating sperm small RNA composition. Together, our results reveal an unexpected role of four epididymis-specific noncanonical ribonuclease A family genes in regulating fertility and small RNA processing.

Sperm released from the testicular seminiferous tubules are not capable of fertilization; they acquire forward motility and fertilization capacity during post-testicular maturation in the epididymis (1). The epididymis is anatomically and functionally subdivided into three broad segments: caput (proximal), corpus (middle), and cauda (distal) epididymis. Mature sperm are stored in the cauda epididymis and released upon copulation *via* the vas deferens. Sperm encounter a variety of proteins, lipids, and ions in the epididymal luminal microenvironment, which is shaped by the secretory activity of the local epithelial cells (2, 3, 4, 5). While it is well established that the epididymis plays an essential role in post-testicular sperm maturation and fertility (1, 6, 7), the specific factors in the epididymal microenvironment that regulate fertility are poorly defined.

The epididymis also plays a role in shaping the RNA composition of mature sperm. The RNA payload of mature sperm is composed of a diverse set of small RNA classes, including miRNAs and piwi-interacting RNAs (piRNAs). Two of the most abundant classes of small RNAs in sperm are cleavage products of ribosomal RNAs (rRNA-derived small RNAs or rsRNAs) and fragments of tRNAs (tRFs) (8), also known as tRNA-derived small RNAs (9) or tRNA-derived RNAs (10). Recent studies revealed that sperm undergo dramatic changes in their small RNA payload during epididymal transit, while testicular sperm are highly enriched in piRNAs and mature sperm in the cauda epididymis are enriched in tRFs and rsRNAs (8, 11, 12, 13, 14). We and others reported that a subset of these newly acquired small RNAs are delivered to sperm from epididymal epithelial cells (8, 11, 14, 15, 16, 17, 18, 19). How tRFs and rsRNAs are generated in the epididymal epithelial cells remains unknown. Importantly, sperm small RNAs have been implicated in intergenerational transmission of paternal environmental effects (20, 21, 22, 23, 24). Small RNAs in mature sperm are altered in response to environmental conditions and delivered to the zygote at fertilization, where they can modulate early embryonic development. Hence, it is crucial to elucidate the biogenesis of sperm small RNAs, as it may be a target of signaling pathways that link paternal environment conditions to offspring phenotypes.

tRNA cleavage has been characterized in several biological contexts, where it is typically induced in response to stress conditions. In *Saccharomyces cerevisiae*, *Tetrahymena thermophila*, and *Arabidopsis* *thaliana*, RNase T2 family endonucleases process tRNAs (25, 26, 27), whereas in mammalian cells exposed to stress, the RNase A family member Angiogenin (encoded by *Rnase5*) cleaves tRNAs (28, 29, 30). RNase A and T2 family endonucleases leave a 2′-3′ cyclic phosphate (2′3′cP) or a 3′ phosphate (3′P) at the 3′ end of RNA, both of which can interfere with small RNA cloning protocols. T4 polynucleotide kinase (PNK) removes cyclic phosphates from the 3′ ends of RNA molecules when the reaction is carried out in the absence of ATP. We previously reported that treatment of sperm total RNA with PNK allows adapter ligation and cloning for deep sequencing of RNAs with a 2′3′cP or 3′P at the 3′ end, revealing an abundant class of 5′ tRFs of 33 to 36 nt. These tRFs are slightly longer than the 28 to 32 nt tRFs sequenced without PNK treatment. The population of longer tRFs was typically generated by cleavage within or immediately downstream of the anticodon. These data suggested that the longer tRFs are likely the initial cleavage products generated by an RNase A or RNase T2 family member at the accessible tRNA loop structures, with the previously described shorter (28–32 nt) fragments representing secondary degradation or trimmed products (8). In addition, we found that PNK treatment resulted in a dramatic increase in the levels of rsRNAs in sperm and that rsRNAs were the most abundant class of small RNAs in sperm (8). Overall, our previous studies revealed that tRFs and rsRNAs are likely generated by RNase A and/or RNase T2 endonuclease in the male reproductive tract. Indeed, recent studies showed that inflammation and stress can induce Angiogenin (31) and RNase T2 (32) expression in the epididymis and increase levels of specific tRFs in sperm. However, robust tRNA cleavage is observed in the epididymis even in the absence of stress (11). The factors involved in the biogenesis of tRFs and rsRNAs in the male reproductive tract under nonstress, physiological conditions remain unknown.

The RNase A superfamily is a vertebrate-specific gene family and consists of 13 members. While proteins within this superfamily share structural and catalytic properties, their primary sequences have undergone considerable divergence, presumably to facilitate the emergence of novel functions (33). RNases 1-8 are known as canonical members due to the presence of conserved catalytic residues and disulfide bonds, and these RNases exhibit diverse expression patterns and catalytic activity (34). Functionally, RNases 1-8 regulate catalytic activity–dependent and catalytic activity–independent processes, including RNA degradation, antimicrobial and antiviral activity, and innate immunity (29, 35, 36, 37, 38). In addition to RNases 1-8, another four RNase A paralogs—RNases 9-12—are highly and specifically expressed in different segments of the epididymis (34, 39, 40, 41, 42, 43, 44). Moreover, *Rnase 9*, *10*, *and 11* are primarily expressed in the principal cells of the epididymis (40, 43) whereas *Rnase12* was reported to be enriched in clear cells (40). Genetic studies of *Rnase10* and *Rnase9* indicate a functional role of these genes in male reproduction. Deletion of *Rnase10* resulted in a male fertility defect in the C57BL/6 strain background but not in CD1 mice, thus showing a strain-specific phenotype (45). *Rnase9* knockout mice were fertile but showed slightly reduced sperm motility immediately after release from the epididymis (46). Additionally, human RNase 9 localizes to sperm heads, and recombinant RNase 9 has antimicrobial activity (44). Whether *Rnase11* and *Rnase12* play a role in male fertility has not been investigated.

RNases 9-12 share 15 to 30% sequence identity with the canonical RNases and retain the signal peptide and three conserved disulfide bonds. Due to a lack of conserved active site sequence motifs, they are predicted to be catalytically inactive and are known as noncanonical members of the RNase A family (34). A bacterial RNase was recently discovered that adopts the same structural folds as Angiogenin and its paralogs but has no sequence similarity with RNase A family members and lacks the conserved disulfide bonds and catalytic triad characteristic of RNase A family (47). This protein was shown to possess potent RNase activity *in vitro* and *in vivo* despite having a catalytic core distinct from that found in the canonical RNase A enzymes. Moreover, Angiogenin has very weak catalytic activity (10^5^–10^6^ times lower than that of RNase 1) but can still cleave tRNAs (48, 49). These observations suggest that RNases 9-12, which are paralogs of Angiogenin, could have catalytic activity and warrant examination of the role of these proteins in RNA processing in the male reproductive tract.

Here, we deleted the genomic cluster on chromosome 14 harboring the *Rnase9, Rnase10, Rnase11, and Rnase12* genes in the FVB mouse strain background. The *Rnase9-12* KO male mice showed complete loss of fertility as they failed to generate pups when mated with WT females. The KO sperm did not fertilize oocyte *in vivo* but were capable of fertilizing oocytes *in vitro*. While KO sperm did not show any defects in morphology and number, they had motility defects, showed loss of surface peptidase protein ADAM3, and did not form clusters. All these defects can result in an inability of sperm to traverse the utero-tubular junction (UTJ). Indeed, our results show that KO sperm likely cannot reach the oocytes to fertilize due to an inability to traverse the UTJ. We found that the epididymis of KO mice had transcriptomic and proteomic changes consistent with fertility defects. Finally, we investigated whether these proteins are involved in RNA processing in the epididymis. Interestingly, tRFs and rsRNAs were downregulated in the epididymis and sperm of KO mice, demonstrating that *Rnase9-12* play a role in regulating small RNA levels in the epididymis and, thus, modulate the repertoire of small RNAs in mature sperm.

## Results

### Deletion of *the Rnase9-12* gene cluster resulted in the loss of male fertility

**Figure 1:**
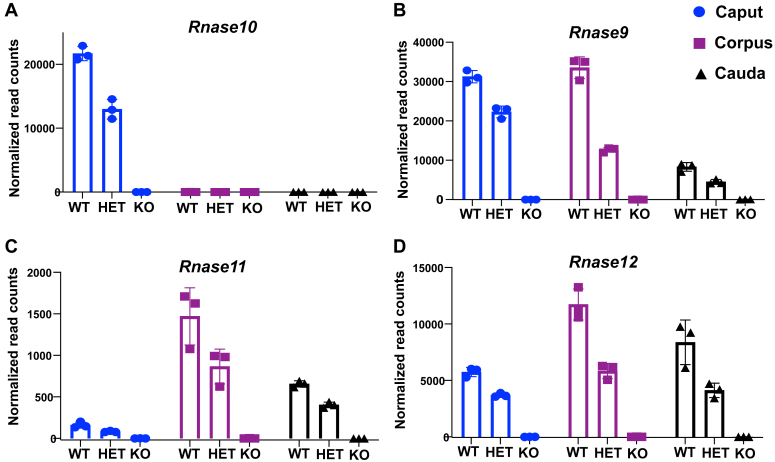
**Generation of mice with deletion of a genomic locus harboring four reproductive tract–specific RNase A family genes.** Normalized read counts of *Rnase10* (*A*), *Rnase9* (*B*), *Rnase11* (*C*), and *Rnase12* (*D*) from mRNA-seq performed on the caput, corpus, and cauda epididymis tissues of WT, heterozygous deletion (HET), and homozygous deletion (KO) males. Normalized read counts were obtained using DESeq2 analysis, and bar graphs represent read counts from three biological replicates.

To study the role of *Rnase9-12* genes in the epididymis, we generated a mouse line with deletion of the genomic cluster harboring these four genes using CRISPR/Cas9 genome editing (Fig. S1*A*). The heterozygous (*Rnase9-12*^*−/+*^ or HET) and homozygous (*Rnase9-12*^*−/−*^ or KO) deletion mice showed the expected decrease in the levels of transcripts of these genes in the epididymis when compared to WT (*Rnase9-12*^*+/+*^ or WT), as seen by mRNA sequencing and quantitative real time-PCR (Figs. 1, *A*–*D* and S1*B*). These data confirmed a complete knockout of the *Rnase9-12* locus. Moreover, our mRNA-seq analysis corroborated previous studies reporting segment-specific expression of *Rnase9-12*, with *Rnase10* being expressed exclusively in the caput epididymis and *Rnase9* in the caput and corpus epididymis (40, 42, 43, 45). We monitored the genotype of mice in all subsequent experiments to ensure the knockout of all four genes.

**Figure 2:**
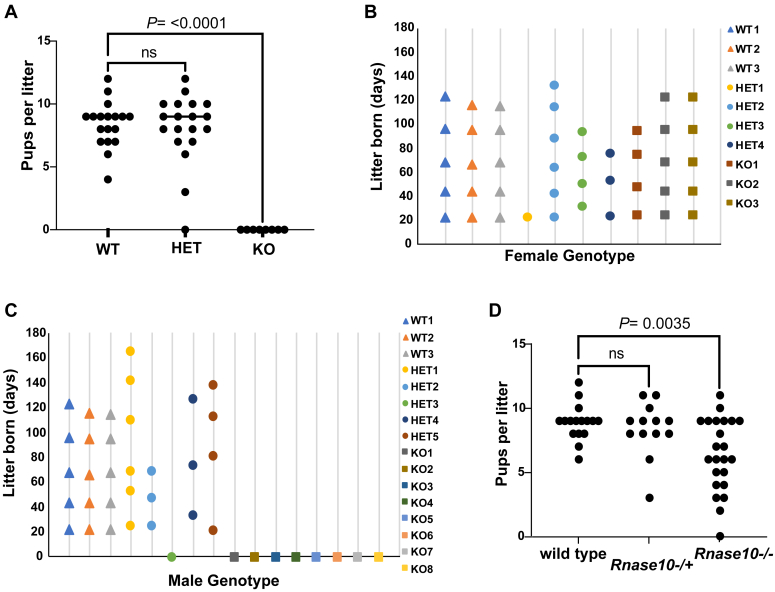
**Loss of male fertility in *Rnase9-12* KO mice.***A*, number of pups obtained per litter from a mating between WT females and males that were either WT, HET, or KO. Each data point represents an independent litter resulting from the following breeding pairs: 3 (WT male X WT female), 5 (HET male X WT female), and 8 (KO male X WT female). *B*-*C*, frequency of litters born days after the start of the breeding cages of females (*B*) or males (C) of WT, HET, and KO genotypes with WT animals of the opposite sex. *D*, number of pups obtained per litter from a mating between WT females and WT males, males heterozygous for a deletion of the *Rnase10* gene *(Rnase10* −/+), or males homozygous for a deletion of the *Rnase10* gene *(Rnase10*^*−/−*^*).* ns (not significant).

At the phenotypic level, the KO mice do not show any differences in physical appearance, behavior, or weight compared to WT mice. Histology of reproductive tract tissues showed no differences in the overall morphology in KO mice compared to WT mice (data not shown). To investigate potential functional roles for these genes in reproduction, we next mated WT, HET, or KO males with WT females and *vice versa*. In matings with WT females, WT and HET males produced normal numbers of pups, while KO males failed to produce any pups; in matings with WT males, females of all three genotypes produced normal numbers of pups (Fig. 2, *A*–*C*). Over 4 to 6 months, the KO males did not produce any litters (n = 8 males). In contrast, HET males showed normal reproductive capacity, demonstrating the haplosufficiency of these four genes. These data indicate that the *Rnase9-12* genomic cluster is required for male fertility.

A previous study reported that *Rnase10* deletion in the C57BL/6 strain background, but not in the CD1 outbred strain, leads to fertility defects (45). The loss of fertility in KO mice in the FVB background (generated in the current study) might be due solely to the deletion of the *Rnase10* gene. We, therefore, generated mice with *Rnase10* deleted (*Rnase10*−/−) in the FVB background (Fig. S1*C*). *Rnase10*−/− males produced fewer pups per litter but did not show complete loss of fertility as observed in the KO mice (Fig. 2*D*). These data demonstrate that the sterile phenotype of the *Rnase9-12* KO males is not simply a consequence of the loss of the *Rnase10* gene and that additional genes from the *Rnase9-12* cluster impact fertility.

### *Rnase9-12* KO sperm are defective in fertilizing oocytes *in vivo* but capable of fertilizing oocytes *in vitro*

**Figure 3:**
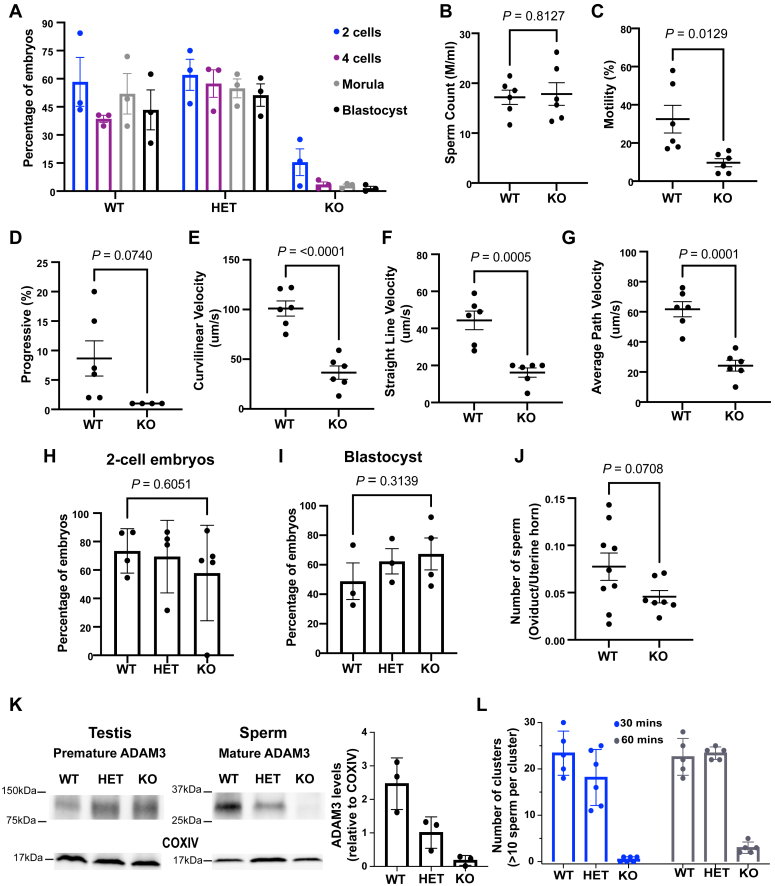
***Rnase9-12* KO sperm are incapable of fertilizing oocytes *in vivo*.***A*, percentage of embryos at specific preimplantation developmental stages. WT females were mated with WT, HET, or KO males overnight, and oocytes were collected from females that showed copulatory plugs the next day. The fertilized oocytes were allowed to develop *in vitro* to the blastocyst stage, and embryos at each stage were quantified as the percent of oocytes that reached the specific developmental stage. The experiment was performed with three males per genotype and 7–9 total females per genotype. *B*–*F*, computer-assisted sperm-analysis (CASA) of WT and KO sperm including total sperm counts (*B*), percentage of motile sperm (*C*), % progressive sperm (*D*), curvilinear velocity (*E*), straight-line velocity (*F*), and average path velocity (*G*). The *p*-values were calculated using unpaired *t* test. *H*–*I*, oocytes from WT females were *in vitro* fertilized using sperm from WT, HET, or KO males. The plots show the percentage of oocytes that developed to the 2-cell embryo stage (*H*) and the percentage of 2-cell embryos that reached the blastocyst stage (*I*). *J*, number of sperm in the oviduct as a fraction of total sperm in the uterine horns. Females were housed with WT or KO males, and 1 h after a copulatory plug was observed, oviducts and uterine horns were dissected to count the number of sperm. A few females that were housed with KO males did not show a copulatory plug and no sperm were detected in their uterine horns. Those females were not included in further analysis. *K*, Western blot analysis of mature ADAM3 protein levels in WT, HET, and KO sperm. A premature form of ADAM3 was detected in the testis (∼100 kDa), and mature ADAM3 was detected in cauda sperm (∼30 kDa). COXIV was used as a loading control. The bar graph represents the quantification of ADAM3 levels relative to COXIV in sperm from three independent biological replicates. *L*, bar graph depicting the number of sperm clusters observed in the three genotypes. Sperm aggregates (10 or more sperm per cluster) in each microscopic field were calculated in three independent biological replicates. Representative microscopic images are included in Fig. S2.

Failure of KO animals to sire litters could result from defects at multiple stages during reproduction, ranging from inability to fertilize oocytes to failure of embryos to implant to early fetal failure. Therefore, we next examined the fertilization rate of embryos produced by mating WT females with WT, HET, or KO males. Superovulated females were housed with males overnight, and the following day, oocytes were collected from the oviductal ampullae of females that displayed a copulatory plug. The oocytes were cultured *in vitro* to allow the development of fertilized oocytes until the blastocyst stage. Interestingly, while ∼60% of oocytes isolated from females mated with WT and HET males reached the 2-cell stage, only 15% of oocytes derived from mating with KO males reached the 2-cell stage; the remaining cells were primarily unfertilized oocytes. Furthermore, while embryos from WT and HET males progressed well to later stages of development, with approximately 50% reaching the blastocysts stage, most fertilized KO embryos did not progress beyond the 2-cell stage, and only 3% reached the blastocysts stage (Fig. 3*A*). These data showed that KO sperm were defective in fertilizing oocytes during natural mating.

We next investigated the biological basis of the low fertilization rate by sperm from KO males. While the KO sperm counts were comparable to those from WT males (Fig. 3*B*), KO sperm displayed motility defects, including a significant decrease in percentage of motile sperm and reduced curvilinear velocity, straight line velocity, and average path velocity (n = 6) (Figs. 3, *C*–*G* and S2*A*). As sperm motility is important for *in vivo* fertilization, we next examined whether KO sperm can fertilize *in vitro*. To examine the ability of KO sperm to fertilize embryos *in vitro*, we performed *in vitro* fertilization (IVF) using oocytes collected from WT females and sperm collected from WT, HET, or KO males. Interestingly, 2-cell embryos were obtained at comparable rates in all three conditions (there was a lower number of KO sperm-derived 2-cell embryos, but the difference was not statistically significant) (Fig. 3*H*), demonstrating that KO sperm can fertilize oocytes *in vitro* but fail to do so efficiently during natural mating. Furthermore, we did not detect any significant difference in the percentage of blastocysts obtained from IVF using KO sperm compared to WT and HET sperm (Fig. 3*I*). This implies that preimplantation development was not affected in KO sperm-derived embryos generated *via* IVF.

It was previously reported that *Rnase10* deletion mice have fertility defects due to failure in sperm transit through the UTJ (45). Given that KO sperm fertilized oocytes *in vitro* but failed to fertilize *in vivo*, we examined whether KO sperm are defective in migrating through the UTJ. We mated superovulated WT females with either WT or KO males and examined the number of sperm in uterine horns, UTJ, and oviducts of the females that showed copulatory plugs. Consistent with the possibility that KO sperm do not traverse the UTJ efficiently, the oviducts and UTJs of the females that were mated with KO males had fewer sperm than those that mated with WT males (Figs. 3*J* and S2*B*). The inability of sperm to pass through the UTJ can be caused by the loss of ADAM3, a disintegrin and metallopeptidase transmembrane protein, from the surface of cauda epididymal mature sperm (50). ADAM3 is expressed as a precursor protein in the testis and gets processed to generate a smaller mature protein during the epididymal maturation of sperm. While ADAM3 precursor protein levels in the testis were comparable between the three genotypes, KO sperm had no detectable mature ADAM3 protein (Fig. 3*K*). Notably, the levels of ADAM3 were also decreased in HET mice, but the loss was not as dramatic as observed in KO sperm and was sufficient to support fertility. Moreover, KO mice showed a sperm aggregation defect; while WT and HET sperm formed clusters after release from the epididymis, KO sperm failed to form clusters (Figs. 3*L* and S2*C*). Sperm aggregation defects are also observed in *Adam3* (51) and *Rnase10* (45) deletion mice, suggesting that this phenotype is linked to ADAM3 loss. Sperm clustering has been shown to occur in the female reproductive tract and is important for sperm transit through the UTJ (52). Together, these data imply that KO sperm are defective in transiting through the UTJ likely due to reduced motility, loss of ADAM3 from sperm surface, and failure to form sperm clusters (53).

### Transcriptomic and proteomic alterations in *Rnase9-12* KO epididymis tissues

**Figure 4:**
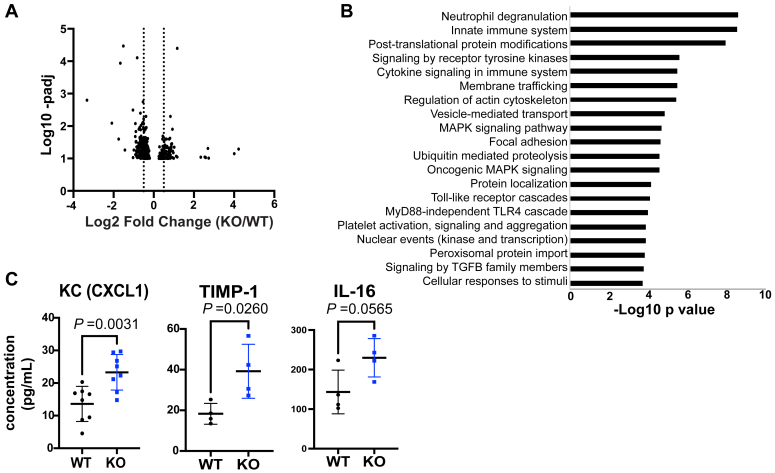
**Transcriptomic and proteomic alterations in *Rnase9-12* KO epididymides.***A*, volcano plot showing mRNA-seq results from DESeq2 analysis of significantly differentially expressed transcripts in the KO cauda epididymis relative to the WT (n = 3 biological replicates). As *Rnase9-12* genes showed the highest and most significant changes in mRNA abundance, those four genes were removed from this plot to make other changes discernable. Volcano plots with all genes in all three regions of the epididymis are included in Fig. S3. *B*, gene ontology Reactome Pathway enrichment analysis of significantly downregulated genes in cauda epididymis tissue. *C*, cytokine and chemokine levels in the cauda epididymal fluid of WT and KO mice.

We performed transcriptome analysis on the epididymis of KO and WT mice to investigate potential pathways affected in KO mice. As expected, *Rnase9-12* were the most significantly downregulated transcripts in KO epididymis tissues than WT tissues (Fig. S3, *A*–*C*). There were 28, 330, and 113 significantly differentially expressed transcripts in the KO caput, corpus, and cauda epididymis relative to WT (*padj* value < 0.05, Log2Fold change >0.5) (Table S1), respectively. In the caput, all 28 misregulated genes were downregulated in the KO. In the corpus, roughly half (167/330) of the misregulated genes were downregulated. In the cauda epididymis, 89 of the 113 misregulated genes were downregulated (Fig. 4*A*). Notably, several downregulated genes in KO epididymides have been previously reported to regulate sperm motility and male fertility, including *Spint4*, *Spint5*, *Crisp1*, *Spinkl*, and *Kcnj16* (Fig. S3 and Table S1) (54, 55, 56, 57).

Gene ontology Reactome pathway analysis of significantly downregulated genes in the cauda epididymis revealed that the top pathways overrepresented were immune system–related, including neutrophil degranulation, innate immune system, cytokine signaling in the immune system, and Toll-like receptor cascades (Fig. 4*B*). In addition, genes involved in translation, cell adhesion, and intracellular transport were over-represented in the differentially downregulated gene set (Fig. 4*B*). To determine if immune processes are altered in KO mice, we examined the levels of cytokines and chemokines in WT and KO cauda epididymides and epididymal luminal fluid. While we did not observe any significant changes in cytokine levels in the epididymis epithelial cells (data not shown), there was an increase in proinflammatory chemokine KC (CXCL1) and cytokines IL-16 and TIMP-1 in KO epididymal fluid compared to WT fluid (Figs. 4*C* and S3*D*).

Proteomic analysis using LC-MS revealed that 109, 39, and 23 proteins were significantly differentially expressed (student’s *t* test, *p*-value<0.05) in the caput, corpus, and cauda epididymis of KO mice relative to WT, respectively (Table S2). Most misregulated proteins were downregulated in the KO epididymis relative to the WT epididymis tissues. Across all three segments of the epididymis, proteins that are part of the 26S proteasome complex were downregulated, including PSMB4, PSMB5, PSMB6, PSMC3, PSME1, and PSME3. Downregulation of the 26S proteasome complex has been associated with male normozoospermic infertility in humans (58) and is involved in proper sperm capacitation and acrosome reaction (59). In addition, proteins involved in the initiation of translation and rRNA processing were downregulated, including RPL23, RPL13, RPL23a, RPL22, RPS28, RPS23, RPS21, and RPLP0. Levels of proteins involved in sperm energy generation and mitochondrial function were also reduced, such as CBR4, DLTA, PDHA2, MLYCD, IDH3A, SUCLG1, UQCRQ, UQCRSF1, COX7A2L, NDUFB8, OXSM, PGLS, ACOX1, ACOX3, L2HGDH, and PDK3. TOLLIP and STAT3, known to have anti-inflammatory functions, were downregulated in KO epididymides. Notably, antioxidants, such as GCLM and NQO1, were downregulated, suggesting that oxidative stress might result in inflammation. These data demonstrate a broad impact of *Rnase9-12* deletion on the biology of the epididymis, including a potential effect on the immune response.

### Altered levels of small RNAs in the epididymis of *Rnase9-12* KO males

RNase A family members have been implicated in the processing/cleavage of noncoding RNAs. For example, Angiogenin cleaves tRNAs to generate tRFs in response to stress (28) and inflammation (31). RNases 9-12 are predicted to be catalytically inactive based on their sequences lacking the RNase A family–specific catalytic residues (34). However, whether RNases 9-12 play a role in RNA processing has not been directly tested *in vivo* except for RNase 10 (60). Cleavage of mature tRNAs and rRNAs leads to the accumulation of tRNA and rRNA fragments (tRFs and rsRNAs) that are detectable by small RNA sequencing. To examine whether tRNA or rRNA processing is affected in the KO mice, we sequenced small RNAs after pretreatment with PNK, allowing us to capture species with 3′ ends (3′P, 2′3′cP) characteristic of RNase A cleavage products (61, 62). Small RNA sequencing reads were analyzed using a modified version of the tRNA Analysis of eXpression (tRAX) analytical pipeline (63) (Experimental Procedures).

**Figure 5:**
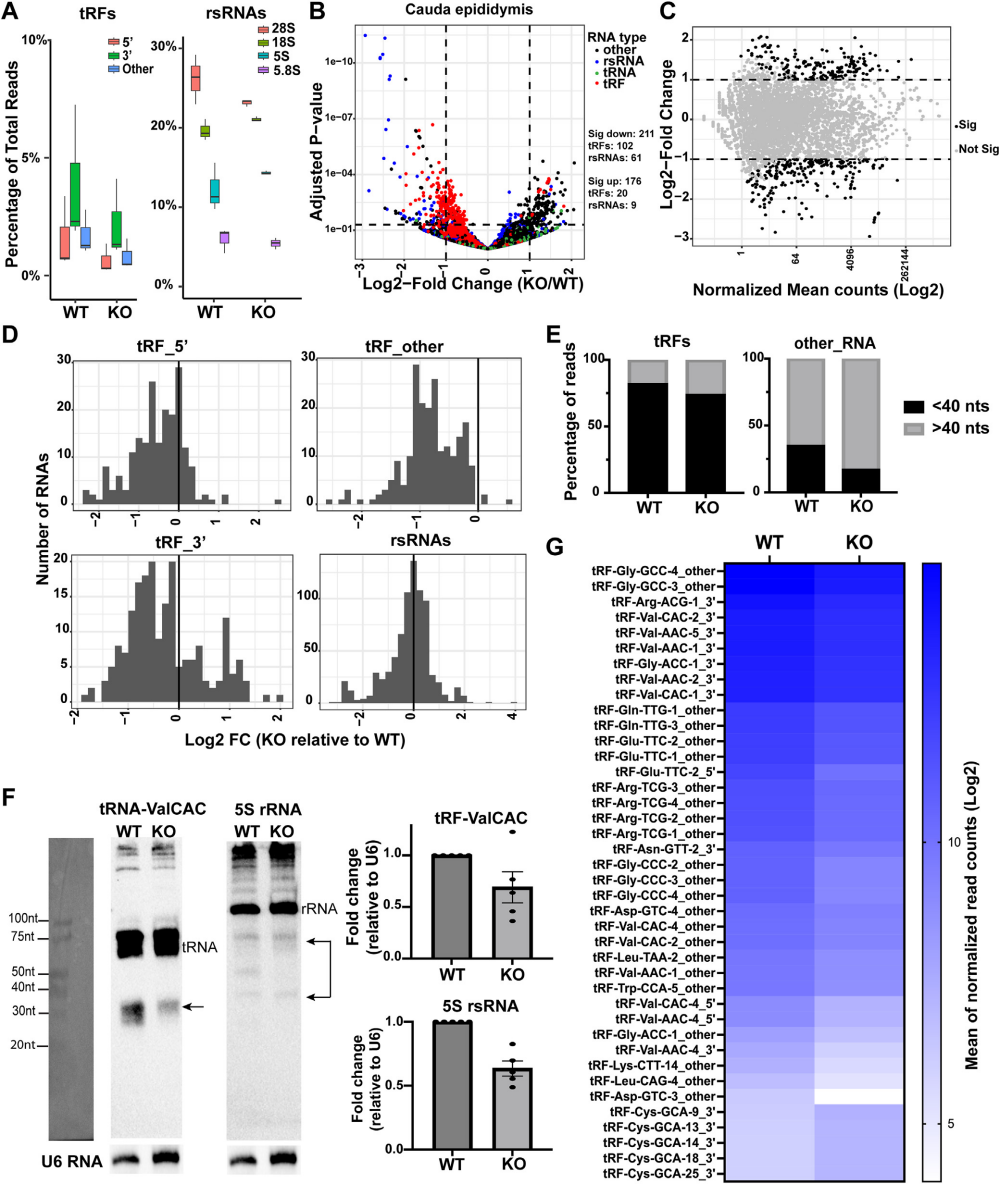
**Small RNA abundance changes in the *Rnase9-12* KO epididymis.***A*, percentage of reads of different subtypes of tRFs and rsRNAs in the cauda epididymis tissues of WT and KO mice (n = 3 biological replicates). The box plots show percentage of all mapped reads (including reads that do not map to any annotated features). The proportion of reads from all annotated RNA types is included in Fig. S4*A*, which shows percentage of reads excluding unannotated reads (accounting for 12–20% percent of mapped reads in different samples). *B*, volcano plot showing differentially expressed small RNAs in the KO cauda epididymis relative to the WT cauda epididymis. tRFs are labeled *red*, tRNAs are *green*, rsRNAs are *blue*, and all other RNAs are *black*. The *dashed* lines show the cut-off used for calling significantly differentially expressed transcripts. The number of significantly differentially expressed transcripts (Log2Fold change >1 and padj value < 0.05) as determined by DESeq2 analysis are shown in the *right* panel. *C*, MA plot showing log2-fold change *versus* normalized mean counts, with significantly differentially expressed RNAs in *black* and nonsignificant RNAs in *gray* (significance cut-off is Log2 fold change of one or more and padj value < 0.05). *D*, histograms showing tRF (tRF_5′, tRF_3′, and tRF_other) and rsRNA abundance changes in KO cauda epididymis relative to the WT. The X-axis shows the log2 fold change of the median of normalized reads, and the Y-axis shows the total count of tRFs or rsRNAs. *E*, percentage of tRF and all other small RNA reads longer than 40 nts (>40 nts) or less than 40 nts (<40 nts). Notably, the KO cauda epididymis showed a higher abundance of >40 nts reads. *F*, Northern blot analysis of fragments derived from tRNA-ValCAC and 5S rRNA. Arrows indicate the fragments that were quantified. For 5S rsRNAs, three different fragments were detected with one of those showing a dramatic decrease in the KO tissues. Shown here is a representative RNA blot sequentially probed in the following order: U6 > tRFValCAC > 5S rsRNA. It should be noted that the same U6 RNA blot image is shown below tRFValCAC and 5S rRNA blots as the same U6 control is used for quantification of these two RNAs. The leftmost gel image is the image of the RNA ladder imaged under a trans-white light setting for the estimation of the molecular weight of the RNA bands. Bar graph shows quantification of tRF-ValCAC and 5S rsRNA levels relative to U6. *G*, heatmap showing log2 mean of normalized read counts of top 40 tRFs significantly differentially expressed between KO and WT cauda epididymides.

Consistent with our previous studies (8, 11), we detected diverse classes of small RNAs in the epididymis epithelial tissue (Fig. S4*A*), with rsRNAs having the highest abundance, consistent with our previous report (8). Interestingly, the KO cauda epididymis tissues showed reduced levels of the proportion of tRF and rsRNA reads compared to WT tissues (Fig. S4*A*), including downregulation of previously characterized functional small RNAs derived from tRNAs and rRNAs such as tRF_5′, tRF_3′, tRF_other, and 28S rsRNAs (9, 11, 13, 64, 65) (Fig. 5*A*). Consistently, differential gene expression analysis revealed that the most significantly altered small RNAs in the KO cauda epididymis relative to the WT were tRFs and rsRNAs, which were mostly downregulated (Fig. 5, *B* and *C*). Examining changes in the abundance of all tRFs (5′, 3′, and other), we observed an overall decrease in the KO cauda epididymis relative to the WT (Fig. 5*D*). Further examination of subtypes of rsRNAs revealed that rsRNAs derived from rRNA repeats (66, 67) were most significantly differentially expressed between WT and KO tissues. Northern blot analysis also revealed reduced levels of a 5′fragment of tRNA-Valine and specific rsRNAs derived from 5S rRNA in KO cauda epididymides (Fig. 5*F*). Moreover, we observed an overall increase in longer (>40 nts) RNA molecules for both tRF reads and all other small RNA (primarily rsRNA) reads in the KO cauda epididymides compared to WT (Fig. 5*E*). Although the small RNA sequencing method used here only captures a fraction of full-length tRNAs, consistent with the hypothesis that the increased levels of longer RNAs in KO cauda epididymides are due to less tRNA cleavage, we detected subtle upregulation of full-length tRNAs in the KO cauda epididymides (Figs. 5*B* and S4*D*). Focusing on tRFs, we found that numerous tRFs previously described to be highly abundant in the epididymis and mature sperm were downregulated, including fragments of tRNA-Gly-GCC, tRNA-Gly-CCC, tRNA-Val-CAC, tRNA-Val-AAC, tRNA-Gln-TTG, and tRNA-Glu-TTC (Fig. 5*G*) (11, 68). Downregulation of numerous tRFs and rsRNAs was also observed in the caput and corpus epididymis, albeit fewer small RNAs showed significant changes in these tissues (Fig. S4, *B* and *C*). Together, these data demonstrated that KO epididymides had reduced levels of tRFs and rsRNAs, suggesting that loss of the *Rnase9-12* gene cluster causes reduced biogenesis and/or reduced stability of those two classes of small RNAs.

**Figure 6:**
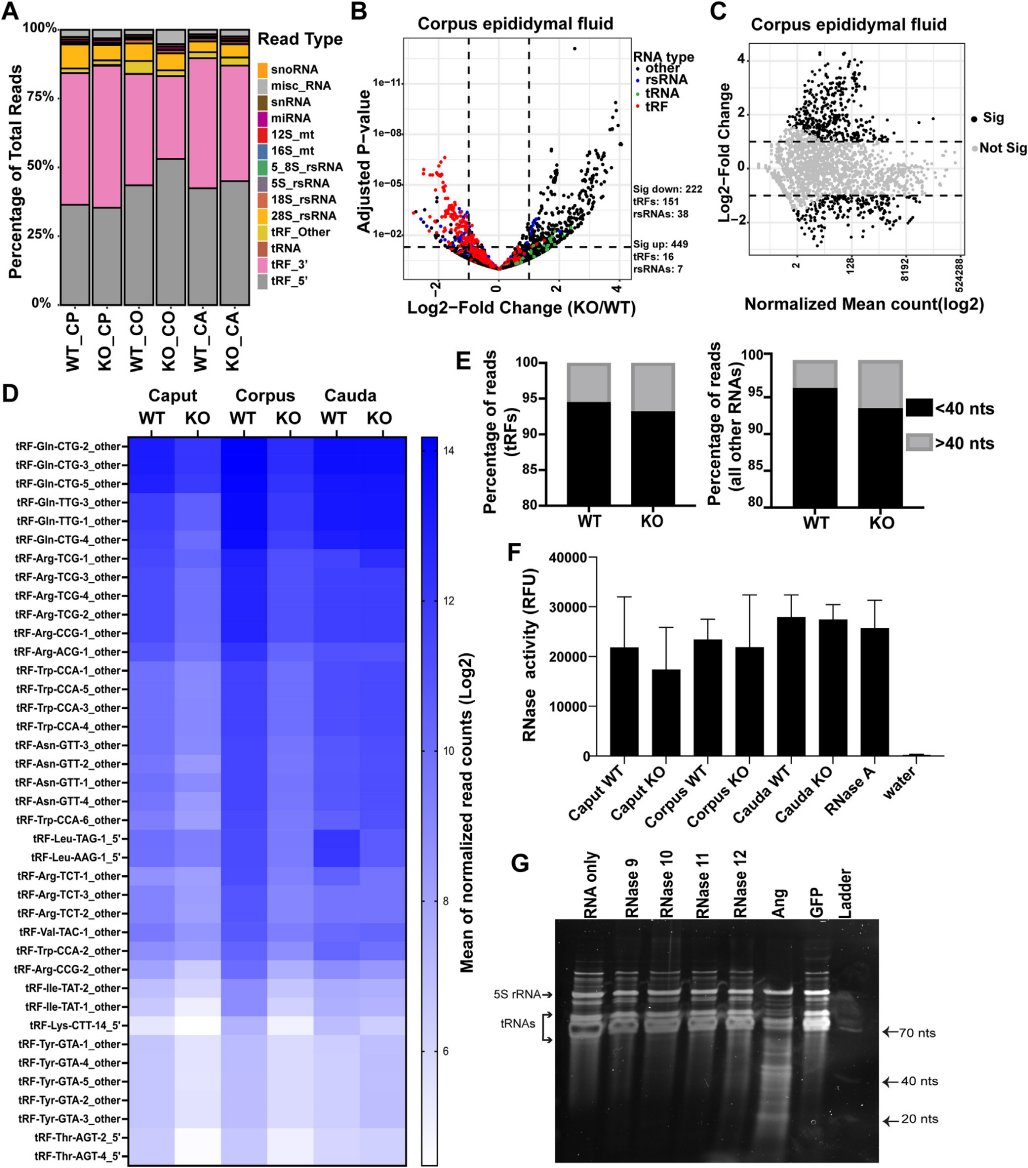
**Reduced levels of tRFs in the epididymal luminal fluid of *Rnase9-12* KO mice.***A*, percentage of reads of different RNA classes sequenced from the epididymal fluid (n = 3 biological replicates). tRFs are fragments mapping to tRNA genes and are further classified for downstream analysis as a 5′, 3′, or “other” fragment based on the read alignment with respect to the ends of the respective tRNA. rsRNA subunits are reads mapping to the specific rRNA subunit and the repeats of that subunit (see Experimental Procedures). ∼7 to 12% of reads did not map to any annotated gene features (“Other” reads) and were removed before generating this plot. The epididymal fluid had a high abundance of reads mapping to tRFs (>85%). *B*, volcano plot showing differentially expressed small RNAs in KO corpus epididymal fluid relative to WT. tRFs are labeled *red*, tRNAs are *green*, rsRNAs are *blue*, and all other RNAs are *black*. The *dashed* lines show the cut-off used for calling significantly differentially expressed transcripts. The number of significantly differentially expressed transcripts (Log2Fold change >1 and padj value < 0.05) as determined by DESeq2 analysis are shown in the *right* panel. *C*, MA plot showing log2-fold change *versus* normalized mean counts in corpus epididymal fluid samples, with significantly differentially expressed RNAs in *black* and nonsignificant RNAs in *gray* (significance cut-off is Log2 fold change of one or more and padj value < 0.05). *D*, the heatmap shows the abundance (Log2 mean of normalized read counts) of tRFs in the epididymal fluid across all segments of the epididymis. Top 40 differentially expressed tRFs in the KO corpus epididymal fluid relative to the WT are shown. Notably, most of these tRFs are also downregulated in the KO caput epididymal fluid relative to the WT. *E*, percentage of tRF and all other small RNA reads longer than 40 nts (>40 nts) or less than 40 nts (<40 nts). *F*, RNase activity measurement in the epididymal fluid collected from WT and KO mice (n = 2). Purified RNase A and water were used as positive and negative controls. The bar graph represents mean ± SD. *G*, RNase activity assay using recombinant RNases 9–12. RNA fraction containing tRNAs and 5S rRNAs was isolated from epididymides by size fractionation on a polyacrylamide gel and used for RNase activity assay. Recombinant Angiogenin was used as a positive control.

RNase A family proteins are secreted extracellular proteins. RNase1 was recently shown to regulate levels of extracellular tRFs and Y-RNA fragments (36). Moreover, porcine ortholog of *Rnase10* was identified as the most abundant secreted protein in the proximal epididymis (69). Therefore, we investigated if the extracellular RNA (exRNA) landscape is altered in KO mice. We isolated RNA from epididymal luminal fluid after removing sperm, extracellular vesicles, and any other cellular fractions from the fluid. Small RNA bioanalyzer analysis of exRNAs showed that caput and corpus epididymal fluid contained an almost equal proportion of 20-40 nts and 70-80 nts size RNA peaks. In contrast, cauda epididymal fluid had a higher abundance of 20-40 nts size RNAs (Fig. S5), suggesting increased RNA processing or higher stability of <40 nts size RNAs in the distal cauda epididymal fluid. Intriguingly, small RNA-seq revealed that epididymal exRNAs were highly enriched in tRFs (Fig. 6*A*). Our *in vivo* findings are consistent with recent reports demonstrating that tRFs are typically more stable in cell culture media than most other RNA species (70). Similar to epididymis epithelial cells, differential gene expression analysis revealed downregulation of the levels of specific tRFs and rsRNAs in the KO caput, corpus, and cauda epididymal fluid relative to WT, with the highest number of differentially expressed transcript in the corpus epididymal fluid (Figs. 6, *B* and *C* and S6, *A* and *B*). The top differentially expressed tRFs in the corpus epididymal fluid were downregulated in the KO fluid across all segments (Fig. 6*D*). Moreover, as observed in the epididymis epithelial cells, caput and corpus epididymal fluid displayed increased levels of longer RNAs and full-length tRNAs (Fig. 6*E* and S6*C*). We note that other small RNAs were also significantly altered in the KO epididymal fluid; for example, there was an upregulation of Rny3 and Rn7sk fragments in the KO epididymal fluid (Fig. 6*B* and S6*D*).

Taken together, epididymis epithelium and luminal fluid exRNA small RNA-seq revealed that KO epididymides had reduced levels of specific tRFs and rsRNAs. These results prompted us to examine if RNases 9-12 have catalytic activity and whether the reduction in the levels of tRFs and rsRNAs is due to loss of catalytic activity in the KO epididymis. Epididymal fluid was isolated from WT and KO mice and RNase activity was assessed using an RNase activity detection assay (see **Methods**). We did not detect a significant drop in the overall RNase activity of the KO epididymal fluid compared to WT (Fig. 6*F*), indicating that loss of RNases 9-12 did not decrease the RNase activity of the fluid. As other RNases are likely present in the epididymal fluid and contribute to the RNase activity of the fluid, this assay could not directly test whether RNases 9-12 have catalytic activity. To investigate catalytic activity of these proteins, we purified recombinant RNase 9, RNase 10, RNase 11, and RNase 12 from mammalian cells and performed an *in vitro* RNase activity assay using tRNAs and 5S rRNAs isolated from epididymis epithelial cells as substrates. Angiogenin was used as a positive control as this RNase A family protein is known to cleave tRNAs despite low catalytic activity compared to RNase 1. While Angiogenin-treated tRNAs were efficiently cleaved, we did not detect any cleavage in RNA samples treated with RNases 9-12 (Fig. 6*G*), indicating that these proteins do not have RNase activity (at least *in vitro*). We did not detect reduced expression of any other members of the RNase A family in KO epididymis tissues (in the mRNA-seq and proteomics datasets), implying that the reduced levels of tRFs and rsRNAs in KO epididymides are not due to a decrease in the expression of catalytically active canonical ribonucleases. Alternatively, RNases 9-12 could regulate tRF and rsRNA levels by binding to these small RNAs and stabilizing them. However, we did not observe any selective binding of tRFs and rsRNAs to recombinant RNases 9-12 in *in vitro* pull-down experiments using Myc-tagged recombinant proteins and total RNA isolated from epididymis tissues (Fig. S6*E*). Together, these studies demonstrate that *Rnase9-12* genes regulate tRF and rsRNA levels independent of endonuclease and RNA-binding activities.

### The profile of sperm small RNAs is altered in *Rnase9-12* KO mice

**Figure 7:**
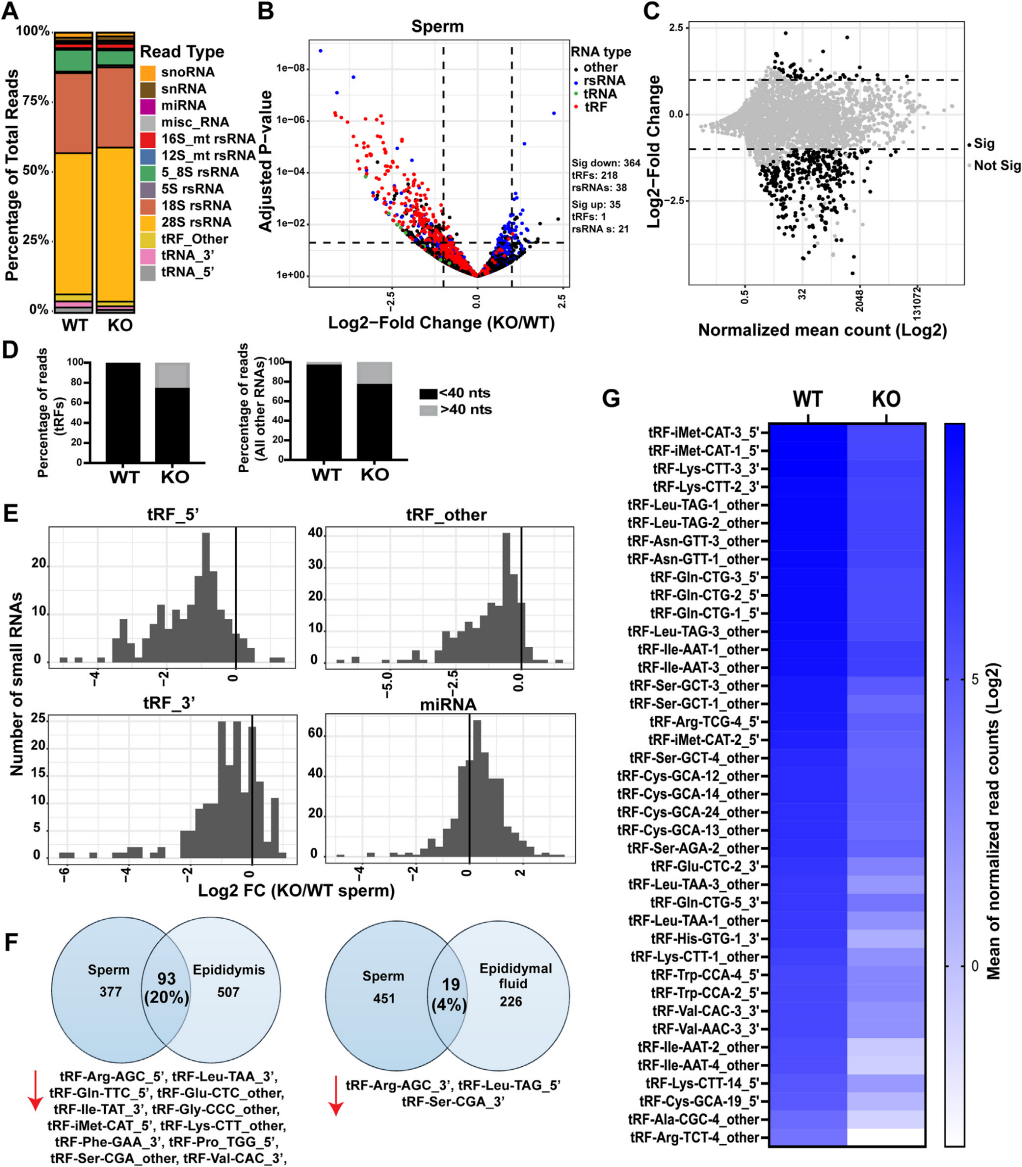
**Changes in small RNA abundance in sperm from*Rnase9-12*KO mice.***A*, read percentage of different classes of small RNAs sequenced from the sperm of WT and KO mice. Approximately 50% of reads did not map to any annotated gene features (“Other” reads) and were removed before generating this plot (n = 4 biological replicates per genotype). *B*, the volcano plot shows differentially expressed small RNAs in the KO sperm relative to WT. tRFs are labeled *red*, tRNAs are *green*, rsRNAs are *blue*, and all other RNAs are *black*. The *dashed* lines show the cut-off used for calling significantly differentially expressed transcripts. The number of significantly differentially expressed transcripts (Log2Fold change >1 and padj value < 0.05) as determined by DESeq2 analysis are shown in the *right* panel. *C*, MA plot showing log2-fold change *versus* normalized mean counts of sperm samples, with significantly differentially expressed RNAs in *black* and nonsignificant RNAs in *gray* (significance cut-off is Log2 fold change of one or more and padj value < 0.05). *D*, percentage of tRF and all other small RNA reads longer than 40 nts (>40 nts) or less than 40 nts (<40 nts). *E*, tRF (tRF_5′, tRF_3′, and tRF_other) and miRNA abundance change in the KO sperm relative to WT sperm. The X-axis is the log2 fold change of the median of normalized reads, and the Y-axis shows the total number of tRFs or miRNAs. *F*, Venn diagrams showing the number of shared statistically significant (padj value < 0.05) small RNA changes in sperm and cauda epididymis tissues and between sperm and cauda epididymal fluid. The top shared tRFs are listed below the Venn diagrams. *G*, the heatmap shows the log2 mean of normalized read counts of top 40 tRFs, which is significantly differentially abundant tRFs between KO and WT sperm.

Previous studies demonstrated that epididymis shapes the small RNA payload of mature sperm (8, 11, 15, 17, 18, 19, 71). Therefore, we examined the small RNA profile of mature sperm isolated from the cauda epididymis. As previously reported, rsRNAs and tRFs were the most abundant classes of small RNAs sequenced from mature sperm (Fig. 7*A*) (8, 13). Notably, while previous studies using standard ligation-based small RNA-seq primarily sequenced 5′tRFs from only a subset of tRNA isotypes (8, 11), OTTR-seq (see Experimental Procedures) allowed sequencing of tRFs from most isotypes of tRNAs and from 3′ end and middle of tRNAs (tRF_3′ and tRF_other, respectively) (72). Consistent with changes in the epididymis epithelial cells, there was a reduction in the percentage of tRF reads in KO sperm compared to WT sperm (Fig. 7*A*) and an increase in the abundance of >40 nts RNAs (Fig. 7*D*). Differential gene expression analysis revealed that numerous tRFs and a subset of rsRNAs were significantly downregulated in KO sperm relative to WT sperm (Fig. 7, *B*, *C* and *G*). Moreover, the abundance of most tRFs (5′, 3′, and other) decreased in KO sperm relative to WT sperm (Fig. 7*E* and S7, *A*–*C*), implying a global downregulation of tRF levels in KO sperm. Notably, other small RNA classes, such as miRNAs, did not show similar downregulation in KO sperm (Fig. 7*E* and S7*F*). Twenty percentage (93/470) of small RNAs that changed significantly in KO sperm relative to WT sperm were also significantly changed in the cauda epididymis tissue (padj value < 0.05). Furthermore, 23% (88/391) of downregulated small RNAs in KO sperm were also downregulated in the KO cauda epididymis. On the other hand, only 4% (19/470) of significantly differentially expressed small RNAs in sperm were also differentially expressed in the cauda epididymal fluid (Fig. 7*F*). These data indicate that sperm small RNA changes in KO mice are better correlated with small RNA changes in the epididymis epithelial cells and support the possibility that a subset of small RNAs in sperm originate in the epididymis. As epididymis accounts for 20% of small RNA changes in sperm, other pathways of small RNA biogenesis in sperm, including communication with epididymal fluid, cytoplasmic droplet (73), and *in situ* biogenesis, are possible. Together, these studies showed that KO sperm have altered small RNA composition and implicate *Rnase9-12* in regulating sperm tRF levels. Importantly, these studies demonstrate the role of epididymis-expressed RNase A family members in regulating sperm small RNA profile and highlight the significance of sperm maturation in the epididymis in shaping the small RNA payload of mature sperm.

## Discussion

### *Rnase9-12* are required for male fertility

The epididymis is essential for male fertility, but the specific factors and mechanisms underlying this regulation remain poorly understood. Here, we identified the role of four epididymis-specific RNase A family genes in regulating male fertility. Deleting a genomic locus on chromosome 14 harboring the *Rnase9-12* genes resulted in a complete loss of male fertility. As the deletion of a single gene (*Rnase10)* from this cluster resulted in subfertility, the sterile phenotype observed in KO males (*Rnase9-12* deletion) is likely due to a cumulative effect of deletion of two or more genes in this cluster. Similar phenomena have been observed for other gene families (74, 75). For example, deletion of a cluster of beta-defensin genes from chromosome 8 in mice resulted in sterility (76), while deletion of a single defensin gene resulted in only subtle phenotypes (77). Given their segmented expression along the length of the epididymis (Fig. 1)—*Rnase10* is expressed exclusively in the caput, *Rnase9* mainly in caput and corpus, and *Rnase11* and *Rnase12* in the caput, corpus, and cauda epididymis—these genes likely work cooperatively to regulate sperm maturation in the epididymis.

What is the underlying cause of infertility in KO mice? The sperm membrane protein ADAM3 is thought to play a pivotal role in sperm-zona pellucida binding and sperm migration through the UTJ (50, 78). Moreover, sperm clustering has been shown to occur in the female reproductive tract and is important for sperm transit through the UTJ (52). Given that KO sperm had reduced levels of ADAM3 and did not form clusters, our data suggest that deletion of *Rnase9-12* leads to loss of ADAM3 and sperm clustering, resulting in inefficient transit through UTJ. However, as *Rnase10* deletion also resulted in decreased sperm ADAM3 levels and an aggregation defect (45), modulation of additional factors in the KO epididymis likely results in the sterility phenotype observed in *Rnase9-12* KO mice. We found that KO sperm had reduced motility, with significant decrease in curvilinear velocity, straight line velocity, and average path velocity. Motility defects along with inefficient transit through the UTJ can result in an inability of sperm to fertilize oocytes *in vivo*. Consistent with these results, KO sperm failed to fertilize oocytes *in vivo* but were able to fertilize *in vitro*. To investigate the underlying molecular changes in the KO epididymis, we performed transcriptomic and proteomic analyses of the KO tissues relative to the WT. Expression of genes involved in many biological pathways was altered in the KO epididymis, including those involved in regulating fertility.

### *Rnase9-12* regulate levels of tRFs and rsRNAs in the epididymis

RNases 9-12 are members of the vertebrate-specific RNase A superfamily and are paralogous to Angiogenin (42). Angiogenin can cleave tRNAs to generate tRFs in response to stress and inflammation (28, 31). It was recently reported that inflammation can induce the expression of Angiogenin in the epididymis, resulting in increased levels of specific tRFs in sperm (31). However, as robust tRNA cleavage is observed in the epididymis even in the absence of any stressors, other factors likely contribute to tRF biogenesis under nonstress, physiological conditions. Here, we found that deletion of these four epididymis-specific RNase A family members led to a dramatic decrease in the levels of tRFs and rsRNAs in the epididymis and sperm, suggesting a reduced cleavage of their precursor RNA molecules (tRNAs and rRNAs) and/or decreased stability of tRFs and rsRNAs in the KO epididymis. Together, these observations underscore the roles of *Rnase9-12* genes in regulating small RNA levels in the male reproductive tract. It is tempting to speculate that these changes in tRFs and rsRNAs lead to altered gene expression in KO epididymis as these small RNAs have been shown to regulate gene expression at transcriptional and post-transcriptional levels (20).

Numerous studies have reported that tRFs and other noncoding RNAs are abundant in bodily fluids. These extracellular RNAs can be encapsulated in extracellular vesicles and lipoprotein particles or as free-floating RNAs in ribonucleoprotein complexes. Recent reports demonstrated the role of RNase 1 in cleaving tRNAs and Y-RNAs present in the nonvesicular extracellular environment of cultured human cells (36). Since *Rnases9-12* belong to the same family of genes, we investigated their role in processing exRNAs in the nonvesicular fraction of the epididymal luminal fluid. Intriguingly, small RNA-seq revealed that in the epididymal fluid, >85% of reads were from tRFs compared to less than 25% of tRF reads in the epididymis epithelial cells. A high abundance of tRFs in the epididymal fluid suggests that tRNA cleavage takes place in the epididymal fluid to generate tRFs, which can be taken up by the epithelial cells along the length of the epididymis (79). Alternatively, tRFs could be generated in the epididymal epithelial cells and selectively secreted in the epididymal luminal fluid. Notably, KO mice had decreased levels of tRFs in the epididymal fluid exRNAs. The decreased levels of tRFs in the KO epididymal fluid could be due to reduced cleavage of tRNAs in the KO epididymal fluid or a reduction in the levels of tRFs secreted from the epididymis epithelial cells. An important future goal is to elucidate the functional significance of the nonvesicular extracellular tRFs present in the epididymal fluid.

RNases 9-12 are predicted to lack catalytic activity due to the absence of the conserved RNase A family catalytic residues (34), raising the question of how *Rnase9-12* regulate tRF and rsRNA levels. Recently, a bacterial endonuclease was discovered that adopts the same structural folds as Angiogenin and other RNase A paralogs but has no sequence similarity with RNase A and lacks the conserved disulfide bonds and the catalytic triad of RNase family members (47). This protein was shown to possess potent RNase activity *in vitro* and *in vivo* despite having a catalytic core distinct from that found in the canonical RNase A enzymes. These observations suggest that paralogs of Angiogenin that lack RNase A catalytic residues could still have catalytic activity. Our *in vitro* RNase activity assay using recombinant RNases 9-12 indicates that these proteins do not have RNase activity. Consistently, we did not detect a significant drop in the overall RNase activity in the epididymal fluid upon deletion of the *Rnase9-12* genes. Moreover, we did not detect specific binding of tRFs and rsRNAs to RNase 9-12. These data suggest that *Rnase9-12* genes regulate small RNA levels in catalytic activity and RNA-binding property-independent manner. In the absence of catalytic activity, RNases 9-12 could regulate levels of tRFs and rsRNAs by modulating the activity of other RNases or RNase inhibitors. Alternatively, *Rnase9-12* could indirectly regulate levels of tRFs and rsRNAs by regulating pathways involved in the biogenesis and stability of tRFs and rsRNAs. For example, DNMT2 and NSUN2 catalyze 5-methylcytosine modifications, and these modifications can promote tRNA stability by antagonizing stress-induced cleavage by Angiogenin (80, 81). Moreover, loss of DNMT2 leads to alterations of sperm tRF and rsRNA levels, potentially *via* altering the modification status on these RNAs (82). Finally, RNase A family members can be internalized by cells *via* endocytosis or dynamin-independent uptake, where they can regulate cellular processes in a catalytic activity–dependent and catalytic activity–independent manner (83, 84, 85). Future studies with mice expressing epitope-tagged versions of RNases 9-12 will allow direct analysis of their *in vivo* interacting partners to further assess the role of these genes in fertility and small RNA processing. In addition, the link between changes in small RNA levels and sperm motility and transit through the UTJ in the epididymis remains to be elucidated.

### *Rnase9-12* regulate sperm tRF levels

Sperm tRFs have been implicated in regulating early embryonic development and intergenerational epigenetic inheritance of paternal environmental effects (20, 21). For instance, we previously reported that a 5′ fragment of tRNA-Glycine-GCC is upregulated in the sperm of mice exposed to a low-protein diet, and in preimplantation embryos, this tRF regulates the transcription of a subset of genes expressed during zygotic genome activation (11). Recently, a tRF derived from tRNA-Glutamine-TTG was reported to regulate the early cleavage of porcine preimplantation embryos (68). Moreover, various paternal environmental conditions (such as diet, stress, and toxicant exposure) modulate offspring phenotypes, and sperm tRFs are proposed to mediate the inheritance of such paternal environmental effects. For instance, various environmental perturbations alter the levels of tRFs in the sperm of mice (11, 17, 21, 31, 86, 87, 88) and humans (65, 87, 89). Therefore, it is critical to elucidate the biogenesis of sperm tRFs. Here, we uncovered that *Rnase9-12* regulate the small RNA composition of mature mammalian sperm. These genes, thus, could be potential targets of signaling pathways that link paternal environmental conditions to offspring phenotypes. While the KO mice cannot reproduce naturally, a litter could be generated *via* IVF. Irrespective of the underlying mechanism of *Rnase9-12*–mediated regulation of sperm small RNAs, the KO mice provide a unique genetic model to examine the global functions of sperm tRFs in embryonic development and intergenerational transmission of paternal environmental effects, which will be a focus of future studies.

## Experimental procedure

### Animal husbandry and mouse lines

All animal care and use procedures were approved by the University of California Santa Cruz Institutional Animal Care and Use Committee. Mice were group-housed (maximum of 5 per cage) with a 12-h light-dark cycle (lights off at 6 PM) and free access to food and water *ad libitum*. The pups born were weaned at 21 days of age. *Rnase9-12* and *Rnase10* KO mouse lines were generated by Cyagen using CRISPR-Cas9 gene editing. For *Rnase9-12* KO mice, two guide RNAs were used to delete the genomic locus harboring the four genes (Fig. S1*A*). For *Rnase10* KO mice, two guide RNAs were used to delete the 5′ half of exon 2. The KO mice did not show any physical defects. In the *Rnase9-12* line, we occasionally noticed smaller pups and pups with short tails; however, these phenotypes did not correlate with *Rnase9-12* deletion as they were also observed in genotypically WT animals of this line. For genotyping, 200μL of direct PCR lysis reagent (Viagen 102-T) and 2μL of Proteinase K (from 20 mg/ml stock) were added to the tail snip and incubated at 55 °C overnight, followed by boiling at 90 °C for 10 min to isolate the genomic DNA. Next, the microcentrifuge tubes were spun at 12,000*g* for 1 min, and the supernatant was either directly used for PCR or DNA was further purified by ethanol precipitation. BiomixRed (Bioline BIO-25006) PCR mix was used to perform genotyping PCR. The cycle conditions used were as follows: 94 °C for 3 min followed by 35 cycles of 94 °C for 30 s, 60 °C for 35 s and 72 °C for 35 s, and a final extension for 5 min at 72 °C. The PCR products were then run on a 1% Agarose gel to confirm the genotype based on the length of the amplified PCR product.

### Embryo collection after natural mating

Superovulation was induced in 8 weeks old female mice by an intraperitoneal injection of 5IU Pregnant Mare’s Serum Gonadotropin (ProspecBio, HOR-272), followed by an intraperitoneal injection of 5IU human chorionic gonadotropin (hCG) (Millipore Sigma, 230734) 48 h later. Immediately after the hCG injection, females were placed in a cage with males to allow mating. Copulatory plugs were checked 14 h later, and oocytes were collected from the oviducts of females who displayed copulatory plugs 18 h after the hCG injection. Oocytes with cumulus cells were transferred to a 200μL drop of KSOM media containing 3 mg/ml hyaluronidase and incubated for 3 to 4 min. Oocytes separated from cumulus cells were washed and allowed to undergo further preimplantation development in KSOM media in a humidified incubator at 37 °C, 5% CO2, and 5% O2 conditions.

### Sperm agglutination analysis

Cauda epididymis and Vas deferens were collected from the 8 to 12 weeks old WT, HET, and KO males, and sperm were released into prewarmed human tubal fluid (HTF) media containing 0.5% bovine serum albumin and incubated at 37 °C and 5% CO2. Sperm count was carried out and adjusted to 2 × 10^5^ sperm/ml for further analysis. Sperm aggregates (with at least 10 sperm per group) were counted after incubation of sperm for 0, 30, and 60 min in three independent experiments using phase-contrast microscopy.

### *In vitro* fertilization

Cauda epididymis and vas deferens sperm were collected from 8 to 12 weeks old WT and KO males. The tissue was placed in 1 ml prewarmed HTF media containing 0.75 mM methyl-beta-cyclodextrin, and sperm was allowed to swim out by incubating the tissue at 37 °C for 20 to 30 min. Sperm were counted, and 1 × 10^6^ sperm was then pre-incubated for 1 h at 37 °C and 5% CO2 in 200 μl drops of fertilization medium (KSOM + 1 mM Glutathione) covered with sterile mineral oil. Superovulation was induced in female mice using Pregnant Mare’s Serum Gonadotropin and hCG as described above. Cumulus–oocyte complexes were collected from the oviducts of females at 13 to 15 h after hCG injection. These were then transferred to the 200μL fertilization drop containing sperm. The oocytes were then co-cultured with sperm at 37 °C under 5% CO2 for 4 h to allow fertilization. Fertilized zygotes were washed to get rid of excess sperm and cumulus cells and cultured to later stages of development at 37 °C under 5% CO2 and 5% O2.

### Sperm motility assay

Sperm motility was characterized by CASA using iSperm Analyzer M6 (murine from Aidmics Biotechnology). Fresh cauda was collected from 10 weeks old WT and KO mice in 1 ml pre-warmed HTF media in a 35 mm dish. 2 to 3 small cuts were made on the tissue using ultra fine tip scissors and sperm was allowed to flow out along with the cauda epididymal fluid. The dish was incubated at 37 °C for 20 min to allow the cauda to empty itself. The media was then gently mixed and collected in a 1.5 ml microcentrifuge tube leaving the tissue behind. The tubes were then incubated for another 20 min at 37 °C to allow tissue debris to settle down. Twenty microliters of the sperm solution was then loaded onto the iSperm chip and the chip was mounted to the lens attached to an iPad mini. The murine iSperm CASA software was used to analyze the samples and the data was later exported and used to make the plots. The tested parameters include sperm count, motility, progressive percent, curvilinear velocity, average path velocity, straight line velocity, straightness percent, and linearity percent (n = 6 males of each genotype were analyzed).

### Analysis of sperm counts in UTJ and oviduct

Experiments were performed as previously reported (90, 91). Superovulation was induced in 8 weeks old WT female mice as described above for IVF. Twelve hours after HCG injections, two females were housed with one WT or KO male for mating and were checked for the formation of vaginal plug every 30 min. Two hours after copulation (as indicated by the presence of a copulatory plug), the females were sacrificed and the uterine horn and oviduct were carefully separated and put in separate 1.5 ml microcentrifuge tubes containing 500μL of prewarmed KSOM media. The tissue was gently chopped using fine tip scissors inside the tube and incubated at 37 °C for 20 to 30 min. Ten microliters of the solution were mounted on a Neubauer Chamber (hemocytometer) and the number of sperm in uterine horns and oviducts were counted. The values were used to calculate the number of sperm in the oviduct relative to the total number of sperm in the respective uterine horn from the same female. Atleast seven females were analyzed in each group.

To visualize sperm in the oviducts of females mated with WT or KO males, the dissected uncoiled oviduct and uterine horn were frozen in optimal cutting temperature compound to make cryomolds for sectioning. Ten micrometer sections were cut on a cryostat for H&E staining. Prior to staining, slides were washed in 1x PBS to clear optimal cutting temperature. Staining was completed using Abcam’s H&E Staining Kit (ab245880). Briefly, slides were immersed in Hematoxylin for 5 min, quickly submerged in Bluing Reagent for 15 s, and dipped in 100% ethanol. Next, the sections were counterstained in Eosin for 3 min, then dehydrated in three changes of 100% ethanol. Sections were covered in Prolong Gold antifade reagent, and a coverslip was sealed with clear nail polish. Slides were allowed to dry overnight before viewing under a widefield microscope (Leica Widefield). Images of the UTJ were captured at 10X and 40X for viewing of sperm cells. Sperm was identified as cells having a hook-shaped head.

### Tissue collection

Epididymis tissues and cauda sperm were collected as described previously (8). For epididymal fluid collection, epididymides were dissected and placed in prewarmed Whitten’s media for 30 min at 37 °C to allow the release of epididymal luminal contents in the medium. After 30 min of incubation, the media containing epididymal luminal contents was transferred to a 1.5 ml tube and incubator for 15 min at 37 °C to allow tissue pieces and any cellular debris to settle. The top fraction (containing sperm and luminal fluid) was transferred to a new tube and spun for 2 min at 8000 rcf to pellet sperm. The supernatant from this spin was spun at 10,000 rcf for 30 min at 4 °C to pellet the remaining sperm and cell debris. The supernatant was then collected and spun at 100,000 g for 2 h at 4 °C to pellet extracellular vesicles. The supernatant from the last spin, that is the epididymal fluid free of sperm, extracellular vesicles, and any cellular debris, was used for small RNA-seq to examine epididymal fluid RNA composition.

### Protein extraction and immunoblotting

Total protein was extracted from caput epididymis, testis, and sperm by homogenizing the tissue/cells in ice-cold RIPA Lysis buffer (G Biosciences) with 1X protease inhibitor cocktail and 1X phosphatase inhibitor cocktail. Sperm was lysed by sonication at 60% power setting for ∼5 s, followed by incubation on ice for 10 to 15 min. Protein was quantified using BCA assay (Pierce BCA Protein Assay Kit). Thirty micrograms of total protein from tissue samples with 4X Laemmli buffer was heat denatured at 99 °C for 5 min and ran on resolving SDS PAGE gel (Biorad 4–20% precast gel #4561096). Sperm protein samples were prepared using a low volume of lysis buffer (∼100 μL), and ∼30 μL was run on the gel. Proteins were transferred to the polyvinylidene fluoride membrane and incubated with a blocking solution (5% skimmed nonfat milk in 1X TBST) for 1 h at room temperature with slow shaking. The membrane was briefly washed with 1X TBST and incubated overnight with the primary antibody dissolved in 1X TBST at 4 °C on slow shaking (ADAM3 from Santa Cruz Biotechnology (sc365288) and COXIV from Cell Signaling Technology (4844s)). The next day, the membrane was washed thrice with 1X TBST (5 min each at RT under rapid shaking conditions) and incubated for an hour with the HRP-labeled secondary antibody in 1X TBST on slow shaking. The membrane was then developed using BioRad Clarity Western ECL substrate and imaged in a chemi-doc (Biorad). The protein bands obtained were quantified using ImageJ. COXIV was used as a loading control for normalization and quantification.

### mRNA-sequencing and data analysis

Epididymis segments were collected as described previously (8). An Illumina TruSeq Stranded mRNA library preparation kit was used to generate mRNA-seq libraries. The libraries were sequenced on HiSeq, and differential gene expression analysis was performed using the DESeq2 analytical tool (92).

### Quantitative real-time PCR

RNA was extracted using Tri Reagent (Sigma T9424-200 Ml), followed by chloroform-assisted phase separation and precipitation of RNA from the aqueous phase using isopropanol. Complementary DNA or cDNA was synthesized using Superscript III RT (Thermo Fisher Scientific, 18080093) and random hexamers (Thermofisher SO142). qPCR reactions were performed using KAPA SYBR FAST qPCR mix (Roche KK4601) and custom-designed primers for *Rnase9, Rnase10, Rnase11*, *Rnase12, and Gapdh*.

### Northern blot analysis

Northern Blotting was performed by following the previously described method using nonradioactive probes (93) in conjunction with imaging the blots using SuperSignal West Femto Maximum Sensitivity Substrate (Thermo Fisher Scientific, 34094). Briefly, RNA samples were combined with 2X gel-loading buffer II (Thermo Fisher Scientific AM8546G) and denatured at 95 °C for 5 min, followed by at least 2 min at 4 °C or on ice. RNA samples were run on 15% acrylamide 7M urea TBE gel, and the gel was then stained with 1X SYBR gold for 10 min and imaged on the Bio-Rad Gel Doc XR + machine using the corresponding Image Lab software. The RNA was transferred from the gel to a Nylon membrane (Sigma-Aldrich SIAL-11209299001) at 4 °C in a cold room using transfer stacks (from Trans-Blot Turbo RTA kit, Bio-Rad, 1704272). Next, RNA was cross-linked by incubating at 60 °C for 1 to 2 h in a cross-linking solution. After rinsing the membrane, it was prehybridized with 15 ml of prewarmed Ultrahyb buffer (Thermo Fisher Scientific AM8670) at 37 °C for 30 min. The specific LNA probe was then denatured at 95 °C for 1 min and added to the hybridization buffer. The membrane was hybridized overnight on rotation. Next, the membrane was washed twice at 37 °C for 15 min with 2X SSC with 0.1% (wt/vol) SDS, followed by two washes at 37 °C for 5 min with 0.1X SSC with 0.1% (wt/vol) SDS. The membrane was next washed for 10 min with 1x SSC at 37 °C. The membrane was next treated using a Chemiluminescent Nucleic Acid Detection Module Kit (Thermo Fisher Scientific catalog #89880, 24 ml) and imaged with the SuperSignal West Femto Maximum Sensitivity Substrate (Thermo Fisher Scientific Cat# 34096) kit and the Bio-Rad ChemiDocTM MP Imaging System. The same membrane was used to probe other target RNAs after stripping and rehybridizing in the same manner as described above.

### Proteomic analysis

Caput, corpus, and cauda epididymis were harvested from mice and stored at −70 °C after flash freezing in liquid nitrogen. The tissues were taken out of −70 °C and immersed in lysis buffer (1% SDS, 50 mM Tris pH8.1, 10 mM EDTA pH8, protease inhibitor cocktail) and ground with a drill and disposable tips. Tissues were heated in lysis buffer for>2 min at 95 °C to soften the tissue and assist grinding if needed. The protein concentration of lysates was determined using the Pierce BCA Protein Assay Kit (Thermo Fisher Scientific #23225). Ten micrograms of protein from each sample was run on an SDS PAGE gel made using the TGX FastCast Acrylamide Starter Kit, 12% (Bio-Rad #1610174) and using a 1X running buffer made from 10X Tris/Glycine/SDS (Bio-Rad #1610772). Gels were run until the dye front had moved a few centimeters, and then the gels were stained with Bio-Safe Coomassie Stain (Bio-Rad #1610786). Stained proteins were cut out of the gel and shipped to the UC Berkeley Proteomics Core for mass spectrometric analysis.

### RNase activity assay

For RNase activity assay of epididymal fluid, epididymis tissues were dissected from males (10–12 weeks old) and placed in prewarmed Whitten’s media (100 mM NaCl, 4.7 mM KCl, 1.2 mM KH2PO4, 1.2 mM MgSO4, 5.5 mM glucose, 1 mM pyruvic acid, 4.8 mM lactic acid (hemicalcium) and Hepes 20 mM) at 37 °C. A couple of small incisions were made in the epididymis tissues to allow sperm and epididymal fluid to be released from the tissue into the media. Media containing epididymal fluid and sperm was used for protein purification, and total protein content was quantified using the Qubit Protein Assay (Thermo Fisher Scientific Q33211). Nine micrograms of protein was used in each reaction of the RNaseAlert Lab Test Kit (Thermo Fisher Scientific AM1964) to examine RNase activity. Reactions were performed as per the manufacturer’s instructions, and the fluorescence signal (indicative of RNase activity) was measured using the Qubit 4 Fluorometer.

For RNase activity assay using recombinant RNases 9-12, total RNA was Trizol extracted from mouse epididymis and run on a 10% TBE-Urea gel (Biorad 4566033). tRNAs and small rRNAs were size selected from the gel after staining with SYBR Gold (Invitrogen S11494). One microgram of gel-purified RNA was combined with either 0.5, 1.0, or 1.5 μg of recombinant (all with C-Myc/DDK tag) RNase9 (Origene custom product), RNase10 (Origene TP315855), RNase11 (Origene custom product), RNase12 (Origene custom product), Angiogenin (Origene TP308874), or tGFP (Origene TP700079). The volume of each reaction was brought to 15 μl with buffer (100 mM Tris pH 6.60 or pH 6.86, 150 mM NaCl). Reactions were incubated at 5% CO2 and 33 C for > 30 min. Reactions were then mixed with 2X gel loading buffer II (Thermo Fisher Scientific AM8546G) and run on a 10% TBE-Urea gel prior to staining with SYBR Gold and imaging with a Bio Rad Gel Doc XR+. RNase activity assay using three different amounts of protein (0.5μg, 1.0μg, or 1.5μg) showed same results. 1.5μg data is included here.

### RNA-binding assay

Total epididymal RNA was Trizol extracted and either 10 μg or 50 μg of total RNA was incubated with either 1 μg or 5 μg, respectively, of recombinant proteins (all with C-Myc/DDK tag): RNase9 (Origene custom), RNase10 (Origene TP315855), RNase11 (Origene custom), RNase12 (Origene custom), Angiogenin (Origene TP308874), or tGFP (Origene TP700079). Total reaction volumes were brought to either 15 μl or 75 μl, respectively, with RNA-binding buffer (100 mM Tris pH 6.6, 150 mM NaCl). Reactions were incubated at 33 °C and 5% CO2 for >30 min. Reactions were UV cross-linked for 1 min at 450 mJ. Then immunoprecipitation was carried out using 25 μl of Pierce Anti-c-Myc Magnetic Beads, following manufacturer’s protocol. Elution of RNA from the beads was performed in 100 μl buffer (100 mM Tris pH 6.86, 150 mM NaCl) at 55 °C for 10 min with 2 μl of Proteinase k (20 mg/ml) per reaction. Eluates were subsequently Trizol-extracted to purify the RNA. Isolated RNA was sequenced using OTTR-seq.

### Small RNA-seq and data analysis

Small RNA-seq libraries were generated using ARM-Seq (94) or OTTR-seq (62). In both cases, the total RNA was pretreated with PNK to remove 3′ phosphates. For cauda sperm and epididymal fluid samples, total RNA from three mice was pooled to generate one biological replicate, and 1 μg total RNA from the epididymis tissue was used for epididymis libraries. Total RNA was treated with 0.5μL of T4 PNK (NEB M0201 L) at 37 °C for 30 min in a 20μL reaction volume. PNK was heat-inactivated using 0.5μL of 0.5 M EDTA at 65 °C for 15 min, followed by treatment with 3.5μL of 100 mM borax or sodium tetraborate decahydrate for 30 min at 45 °C. Next, the total volume of the reaction was raised to 400μL using nuclease-free water for phenol-chloroform-isoamyl alcohol–mediated RNA cleanup.

Small RNA-seq libraries using OTTR-seq were generated as described by Upton *et al.* (62) and by the manufacturers of the sequencing library preparation kit (Karnateq R2201001S) with a few modifications. Briefly, 40 ng PNK-treated RNA was treated with ddRTP and target primed reverse transcriptase for 2 h, followed by heat inactivation at 65 °C for 5 min. The reaction mixture was next incubated with phosphatase at 37 °C for 15 min, and RNA was reverse transcribed using BoMoc RT (N-terminally truncated *B. mori* R2 Reverse Transcriptase). The cDNA was purified using the MiniElute Reaction Cleanup kit and run on a precast 10% Urea PAGE gel (BioRad #4566033) for 45 min at 200V. cDNA corresponding to small RNAs, including miRNAs and full-length tRNAs, was cut from the gel using Cy5 imaging, and DNA gel extraction was performed. The extracted cDNA was used for PCR reaction for 12 cycles using Q5 Polymerase and NEB multiplex indexing primers (NEB #E7600S). The PCR product was run on a freshly made 6% PAGE gel, and bands corresponding to the expected range of miRNAs to full-length tRNAs (150 bp to 250 bp) were cut out. The eluted PCR product was quantified using a Qubit 1X DNA High Sensitivity kit (Q33230) and analyzed on a Bioanalyzer using a DNA high sensitivity kit (#5067-4626). The final libraries were sequenced either on an Illumina NextSeq instrument.

For sequencing data analysis, sequencing reads were trimmed with cutadapt with adapter “-a GATCGGAAGAGCACACGTCT” and further trimming the final TRPT base with “cutadapt -u -1” and removal of the UMI with “cutadapt -u 7.” Sequencing analysis was done using a modified version of the tRAX analytical analysis tool (63) with default parameters except for a minimum non-tRNA read size of 16 and using the Ensembl gene set (95), the mm10 piRNA gene set from piPipes (96), and ribosomal RNA repeats from UCSC repeatmasker (97) as the gene set. In tRAX, reads were mapped to the mouse mm10 genome combined with tRNA sequences taken from gtRNAdb (98) as default along with additional rRNA sequences. 18S rRNA sequence was taken from NCBI sequence MN537869.1 and 2 28S rRNA sequences were taken from NR_003279.1 and MN537140.1 and 5S rRNA sequence was taken from NR_030686.1 and 5.8S rRNA sequences were taken from NR_003280.2. Mitochondrial rRNA sequences were taken from the Ensembl annotation of the mouse mitochondrial genome. All ribosomal repeat sequences were taken from the UCSC genome browser repeatmasker track for ribosomal RNAs. To create a sequence database for mature tRNA sequences, introns were removed, CCA tails were added, and “G” base was added to the start of histidine tRNAs. tRNA reads were defined as any reads whose best mapping includes a mature tRNA sequence. The tRAX pipeline uses bowtie2 with options "-k 100 --very-sensitive --ignore-quals --np 5 –very-sensitive," extracts all best mappings from those results with some exceptions. For reads that mapped best to tRNAs, the non-tRNA reads were removed and then reads that mapped best to rRNA and repeat sequences mappings to regions other than rRNAs and repeat sequences were removed. tRNA mappings categorizes all tRNA mappings to acceptor type-specific, decoder type-specific, or unique tRNA transcript-specific, and only reads specific to acceptor type and anticodon were used for corresponding tRNA counts. Reads were processed to ensure a single primary read mapping remained and this primary mapping only was used to calculate percentages of total reads for gene types to prevent double-counting.

Reads that mapped to mature tRNAs were further classified into four fragment types based on the read alignment and the ends of the tRNAs. Reads where the 5′ end lies within 3 nts and 3′ end lies with 5 nts of the respective ends on the mature tRNAs are categorized as "whole" full-length tRNAs (referred to as “tRNAs”) and reads that overlap or closely align to either the 5′ ends or 3′ end of the tRNAs are classified as “tRF_5’” and “tRF_3,’” respectively. Mature tRNA reads that do not display these features are classified as “tRF_other.” tRFs_5′, tRFs_3′, and tRFs_other are counted together as “tRFs.” We sequence relatively low levels of full-length tRNAs as the small RNA-seq method favors cloning and sequencing of shorter RNAs (<40 nts). The total percentage of reads for full-length tRNAs was less than 0.5% across all samples and, therefore, not visible on the plots showing the percentage of reads of different small RNA classes. Since small RNA-seq cannot detect RNAs longer than 100 nts, all reads mapping to rRNAs and rRNA-repeats are shorter rRNA fragments and are collectively referred to as rRNA-derived small RNAs (“rsRNAs”). Reads that mapped to the genome but did not map to any annotated feature in either the Ensembl genes, piRNA gene sets, or rRNA repeatmasker were classified as “Other” unannotated read types. Adjusted *p*-values and log2-fold change were calculated using DESeq2 (92) with default parameters as a component of the tRAX pipeline, and plots were generated with ggplot2 and Prism.

## Data availability

The small RNA-sequencing data reported in this study can be accessed at Gene Expression Omnibus with accession number GSE278291. All remaining data are included in the article.

## Supporting information

This article contains supporting information.

## Conflicts of interests

The authors declare that they have no conflicts of interests with the contents of this article.
