# Supplementary material for: Epididymis-specific RNase A family genes regulate fertility and small RNA processing
Source: J Biol Chem. 2024 Oct 28;300(12):107933. doi: 10.1016/j.jbc.2024.107933 (PMC11647505; doi:10.1016/j.jbc.2024.107933)
Supplement: Supporting information [file mmc1.pdf]

Epididymis-specific RNase A family genes regulate fertility and small RNA processing

Joshua F. Shaffer*[1], Alka Gupta*[1], Geetika Kharkwal[1], Edgardo E. Linares[1], Andrew D. Holmes[1], Julian R. Swartz[1], Sol Katzman[2], Upasna Sharma [1†]

[1]Department of Molecular, Cell and Developmental Biology, University of California, Santa Cruz, California, 95064.

[2]Genomics Institute, University of California, Santa Cruz, California, 95064.

[3]Current address: geetikakharkwal@gmail.com

[4]Current address: edgardo.linares@cuanschutz.edu

* These authors contributed equally

[†] To whom correspondence should be addressed: upsharma@ucsc.edu

SUPPLEMENTARY MATERIALS

Supplementary Tables S1-3
Supplementary Figures S1-7

Supplementary Table S1: Transcriptomic alterations in the KO epididymis
Caput, corpus, and cauda epididymis mRNA-Seq data analyzed by DESeq2 are shown. Mean counts, log2 fold change, and *padj* values of significantly differentially expressed transcripts are shown for the caput and corpus (*padj* value <0.05) epididymis and the cauda epididymis (*padj* value <0.1). The data is sorted by *padj* value, lowest to highest.

Supplementary Table S2: Proteomic alterations in the KO epididymis
LC-MS proteomic analysis of WT and KO caput, corpus, and cauda epididymides is shown. For all tissue types, sample 1 is KO, and sample 2 is WT. The data is sorted by p-value, lowest to highest.

Supplementary Table S3: Small RNA-Seq in WT and KO mice. Small RNA-Seq data for epididymides, epididymal fluid, and sperm from WT and KO mice. Total read counts for all examined transcripts are provided here. These raw read counts were normalized as part of the DESeq2 analysis pipeline and used for downstream data analyses.

Supplementary Figure S1

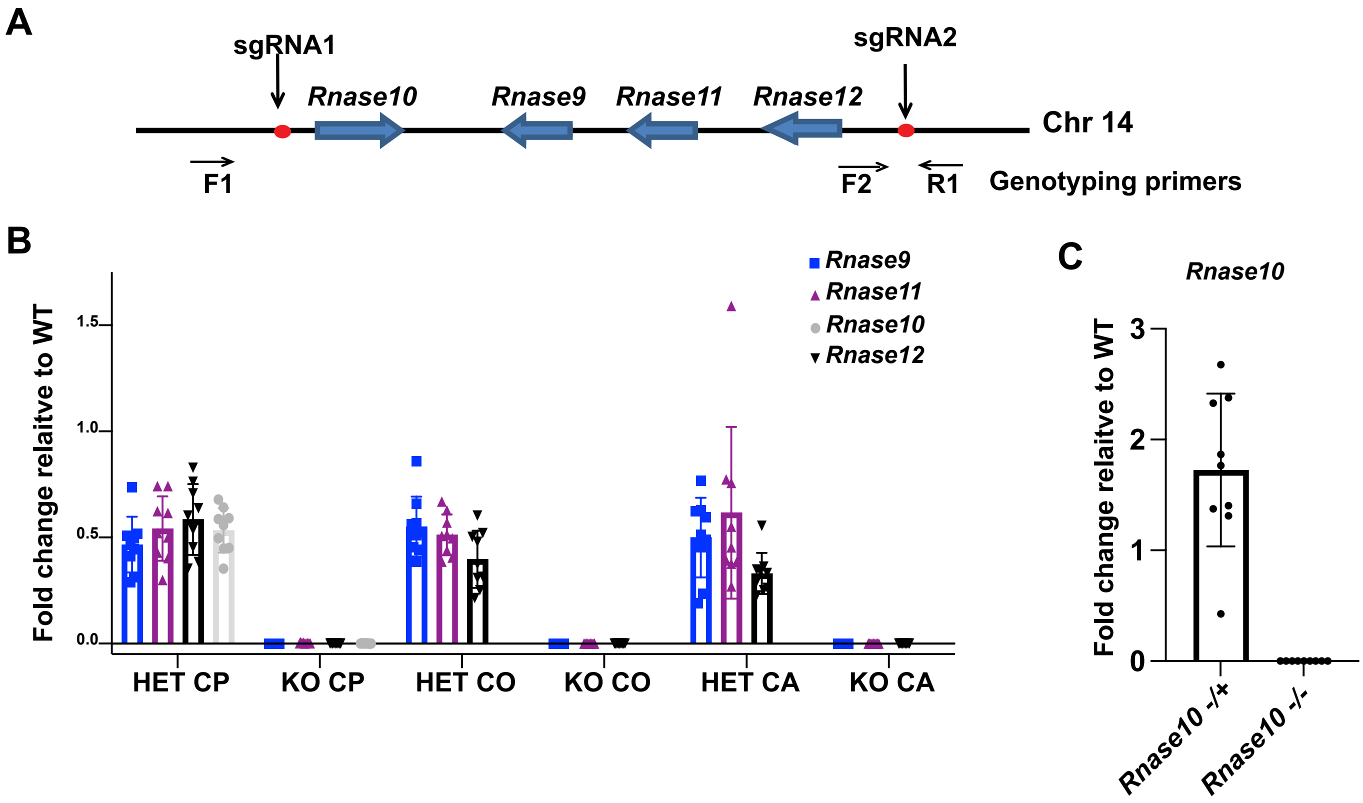

Figure S1: A) Schematic of *Rnase9-12* deletion mouse generation using CRISPR-Cas9 genomic editing. As shown in the schematic, the *Rnase9*, *Rnase10*, *Rnase11*, and *Rnase12* genes are clustered on chromosome 14, and no other protein-coding genes are present in this genomic location. F1-R1 and F2-R1 primer combinations were used to genotype all mice in the study. B) qRT-PCR analysis of *Rnase9, 10, 11*, and *12* transcript abundance in caput, corpus, and cauda epididymides of WT, HET, and KO mice. C) qRT-PCR analysis of *Rnase10* transcript abundance in the caput epididymis of wild-type, *Rnase10-/+* (heterozygous) and *Rnase10-/-* (homozygous) deletion mice. Beta-actin was used for relative quantification.

Supplementary Figure S2

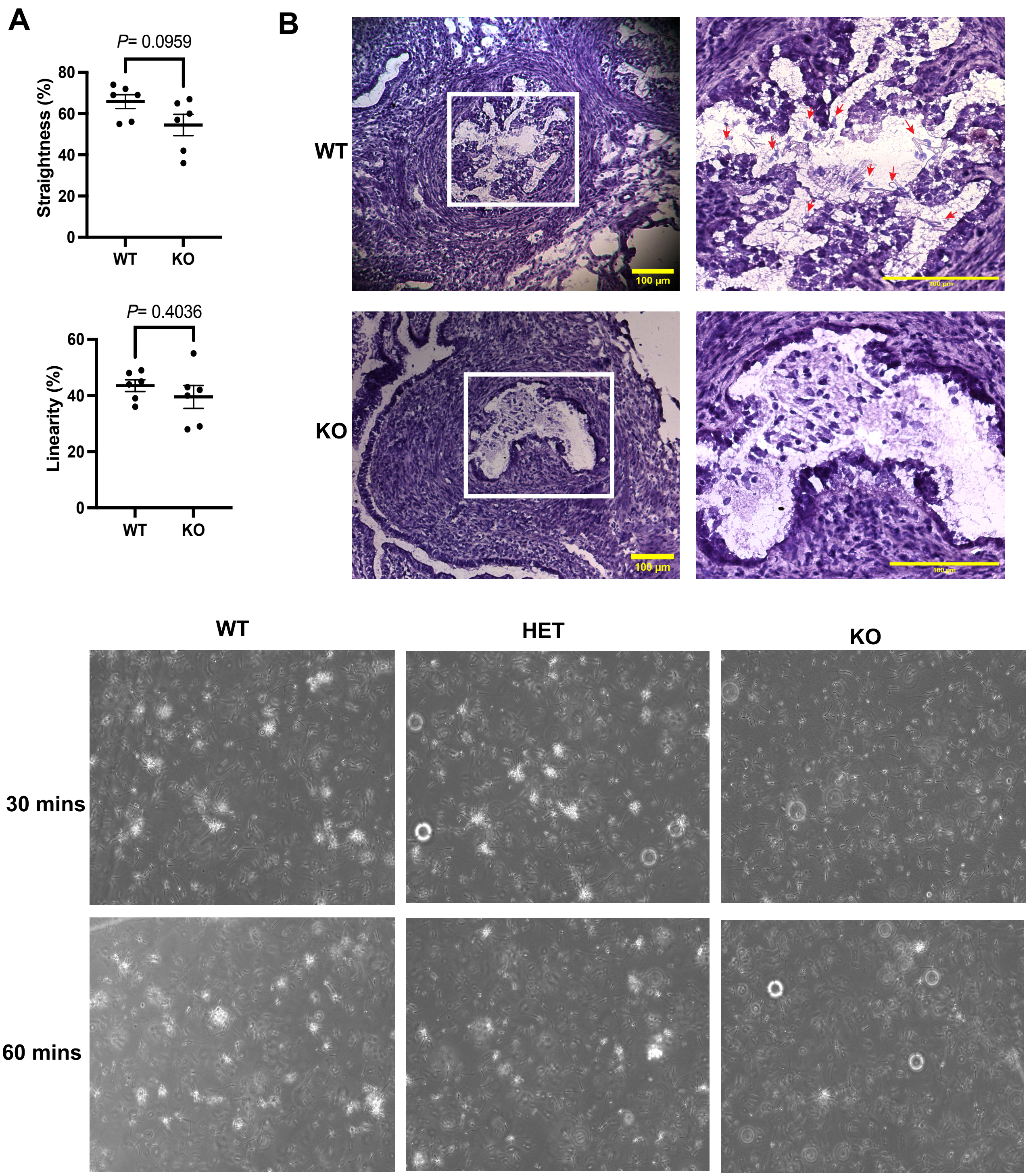

Figure S2: A) Sperm parameters examined by CASA tool. B) H&E-stained cross sections of the UTJ of female mice mated with WT mice or KO mice. The right panels are the rectangle regions at higher magnification from the corresponding left panels. Red arrow indicates sperm. n=2 UTJ sections per male genotype were analyzed. Representative images are shown here. Scale bar represents 100 μm in length. C) Phase contrast microscopy images of WT, HET, and KO sperm. Cauda and vas deferens sperm was released into prewarmed HTF media, and images were taken at 30 and 60 minutes of incubation (n=3 biological replicates per genotype) to examine sperm aggregation.

Supplementary Figure S3

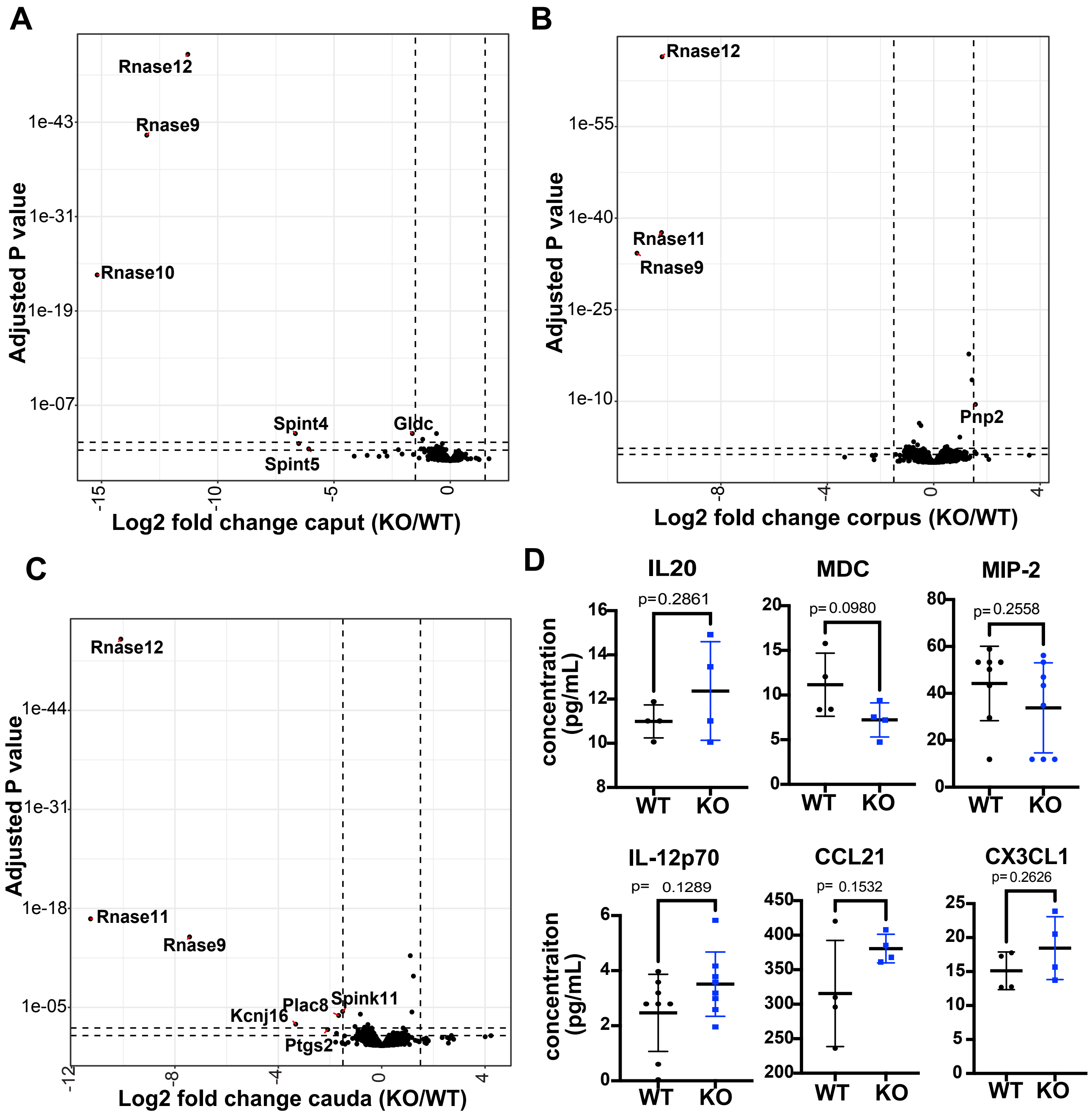

Figure S3: A-C) Volcano plot showing DEseq2 differential expression analysis of mRNA-seq data of KO relative to WT caput (A), corpus (B), and cauda (C) epididymis tissues (n=3 biological replicates per genotype). D) Cytokine and chemokine levels in the cauda epididymal fluid from WT and KO mice (mean ± SD). No significant change was observed for these proteins.

Supplementary Figure S4

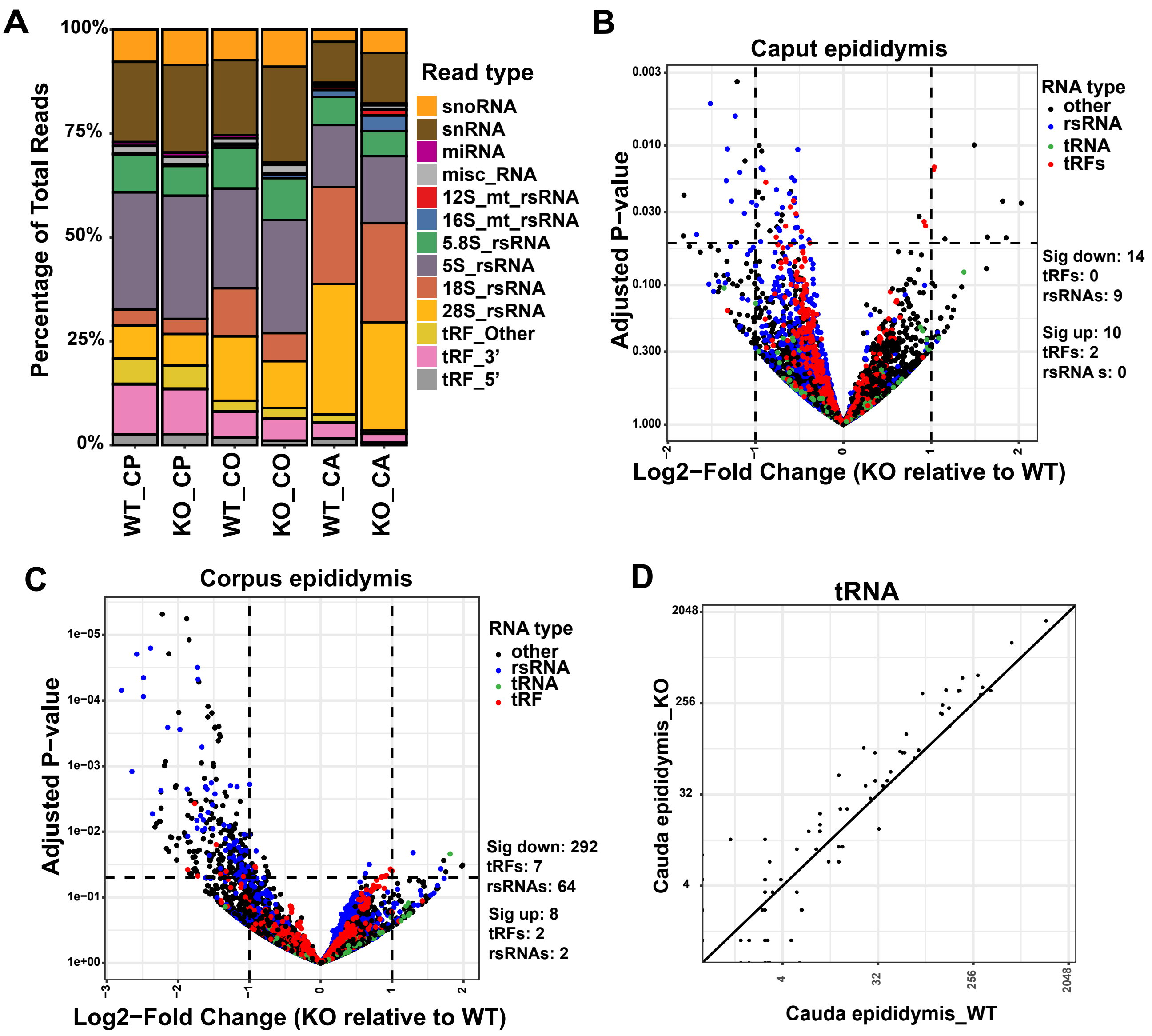

Figure S4: A) Percentage of reads of different small RNA classes sequenced from the caput (CP), corpus (CO), and cauda (CA) epididymis of WT and KO mice (n=3 biological replicates per genotype). tRFs are fragments mapping to tRNA genes and are further classified as a 5', 3', or "other" fragment based on the read alignment with respect to the ends of the respective tRNA. rsRNA subunits are reads mapping to a specific rRNA subunit and the repeats of that subunit (see Materials and Methods). 12-20% of reads did not map to any annotated gene features ("Other" reads) and were removed before generating this plot. B-C) Volcano plot showing differentially expressed small RNAs in the KO caput (B) and corpus (C) epididymis relative to the WT tissues. tRFs are labeled red, tRNAs are green, rsRNAs are blue, and all other RNAs are black. The dashed lines show the cut-off used for calling significantly differentially expressed transcripts. The number of significantly differentially expressed transcripts (Log2Fold change >1 and padj value <0.05) as determined by DESeq2 analysis are shown in the right panel of each plot. D) Scatter plot showing abundance (mean of normalized read counts) of full-length tRNAs in KO and WT cauda epididymides. Although a subtle change, most full-length tRNAs are upregulated in the KO cauda epididymis. These data suggest that there is less tRNA cleavage in the KO cauda epididymides relative to WT.

Supplementary Figure S5

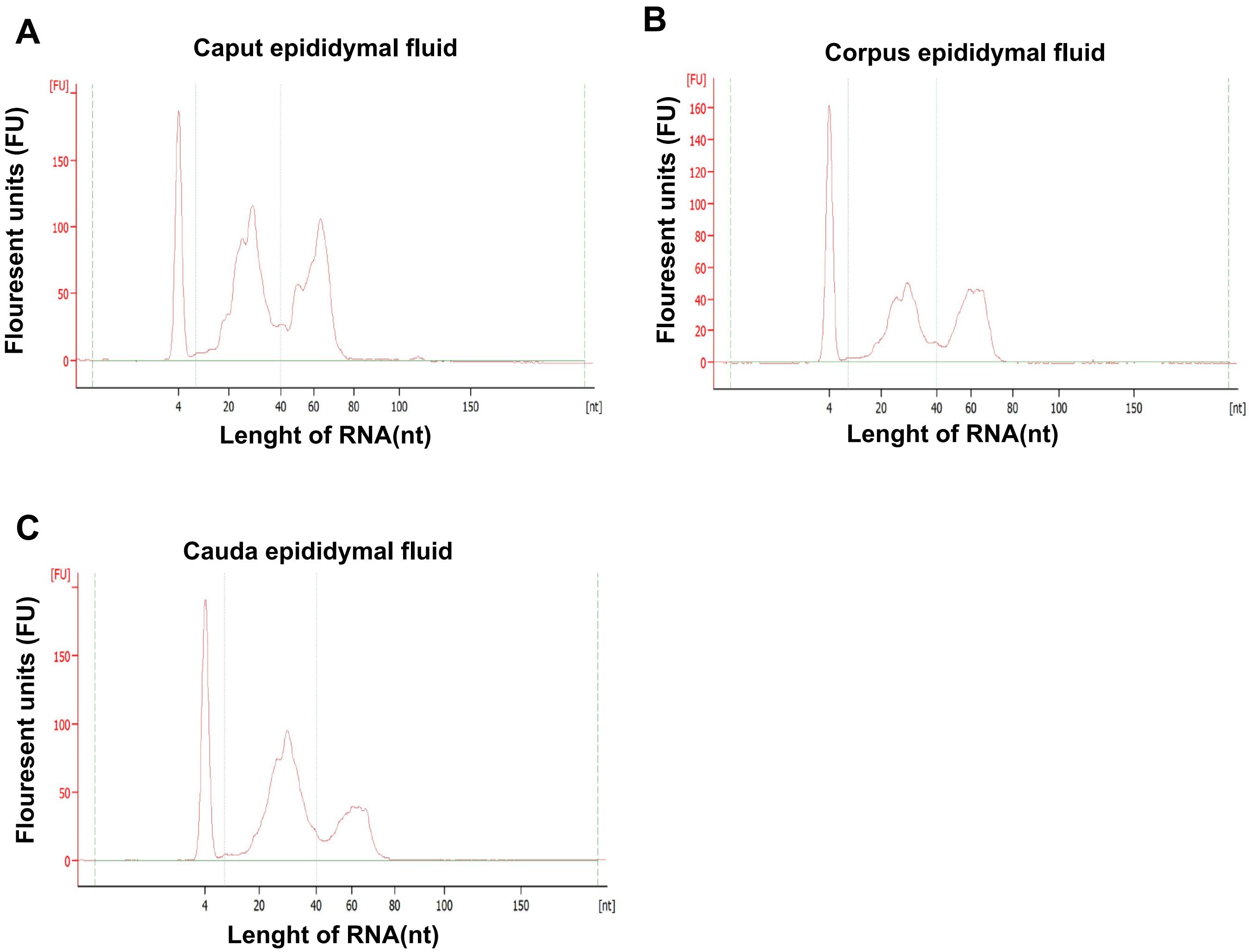

Figure S5: Bioanalyzer trace of total RNA isolated from WT epididymal fluid. The peaks show the length of RNA detected in the epididymal fluid.

Supplementary Figure S6

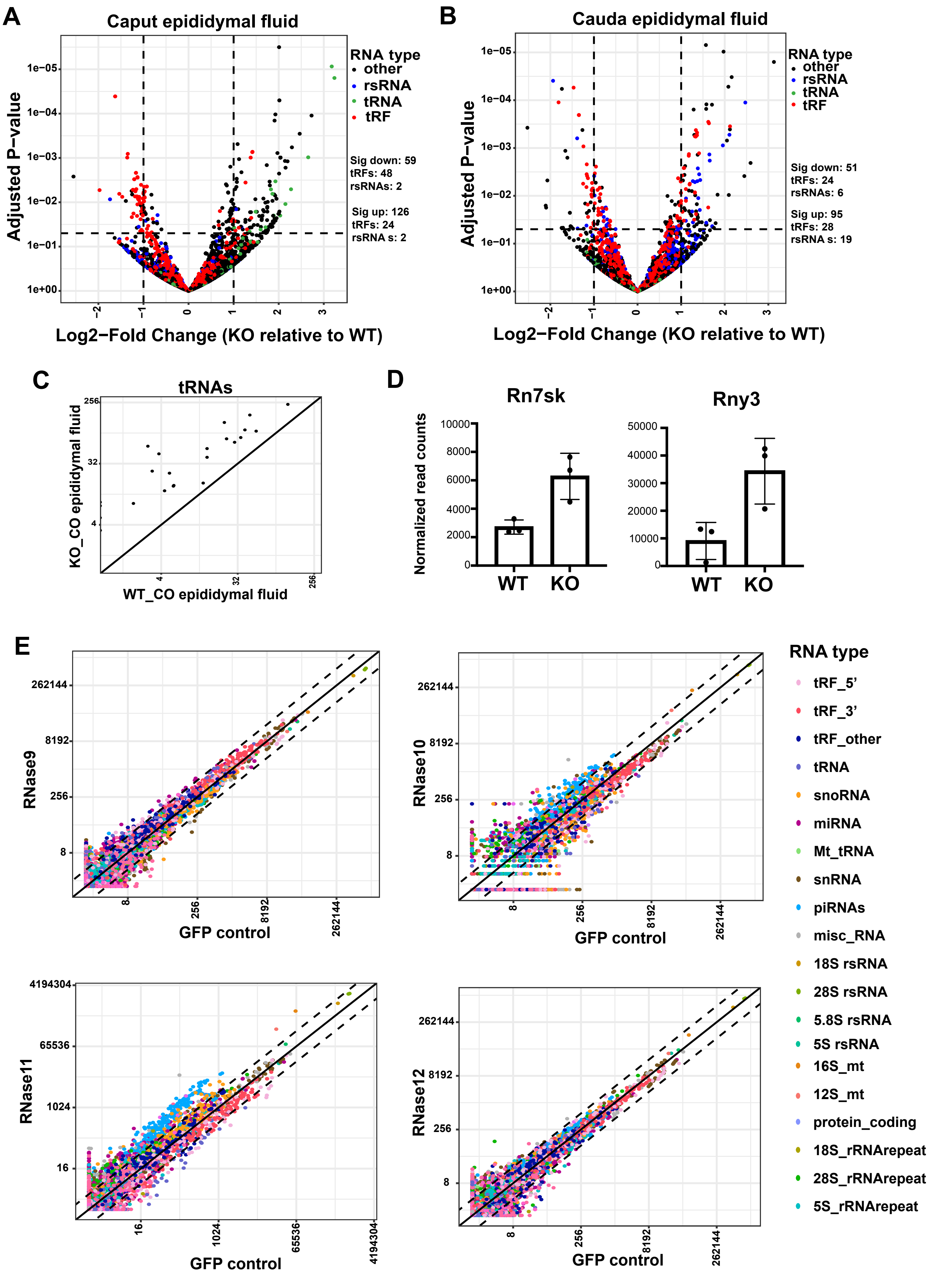

Figure S6: A-B) Volcano plot showing differentially expressed small RNAs in the KO caput (A) and cauda (B) epididymal fluid relative to WT. The dashed lines show the cut-off for calling significantly differentially expressed transcripts (Log2Fold change >1 and padj value <0.05). tRFs are labeled in red, tRNAs in green, rsRNAs in blue, and all other RNAs in black (n=3 biological replicates. C) Scatter plot showing abundance (mean of normalized read counts) of full-length tRNAs in KO and WT corpus epididymal fluid. These data suggest that there is less tRNA cleavage in the KO corpus epididymal fluid relative to WT. D) Normalized read counts of Rn7sk and Rny3 in the caput epididymal fluid. E) Scatter plots of transcript abundance of different small RNAs pulled down with c-Myc-tagged RNases 9-12 versus GFP control (n=2 technical replicates). RNases 9-12 or GFP (negative control) were incubated with total RNA isolated from epididymis tissues. Using Anti-c-Myc magnetic beads RNases and GFP interacting RNAs were pulled down and used for small RNA sequencing. RNase11 pull downs showed slight enrichment of piRNAs. No significant enrichment of tRFs and rsRNAs was detected in small RNAs pulled down with any of the RNases relative to the GFP control, suggesting that these proteins do not bind specifically to these small RNAs.

Supplementary Figure 7

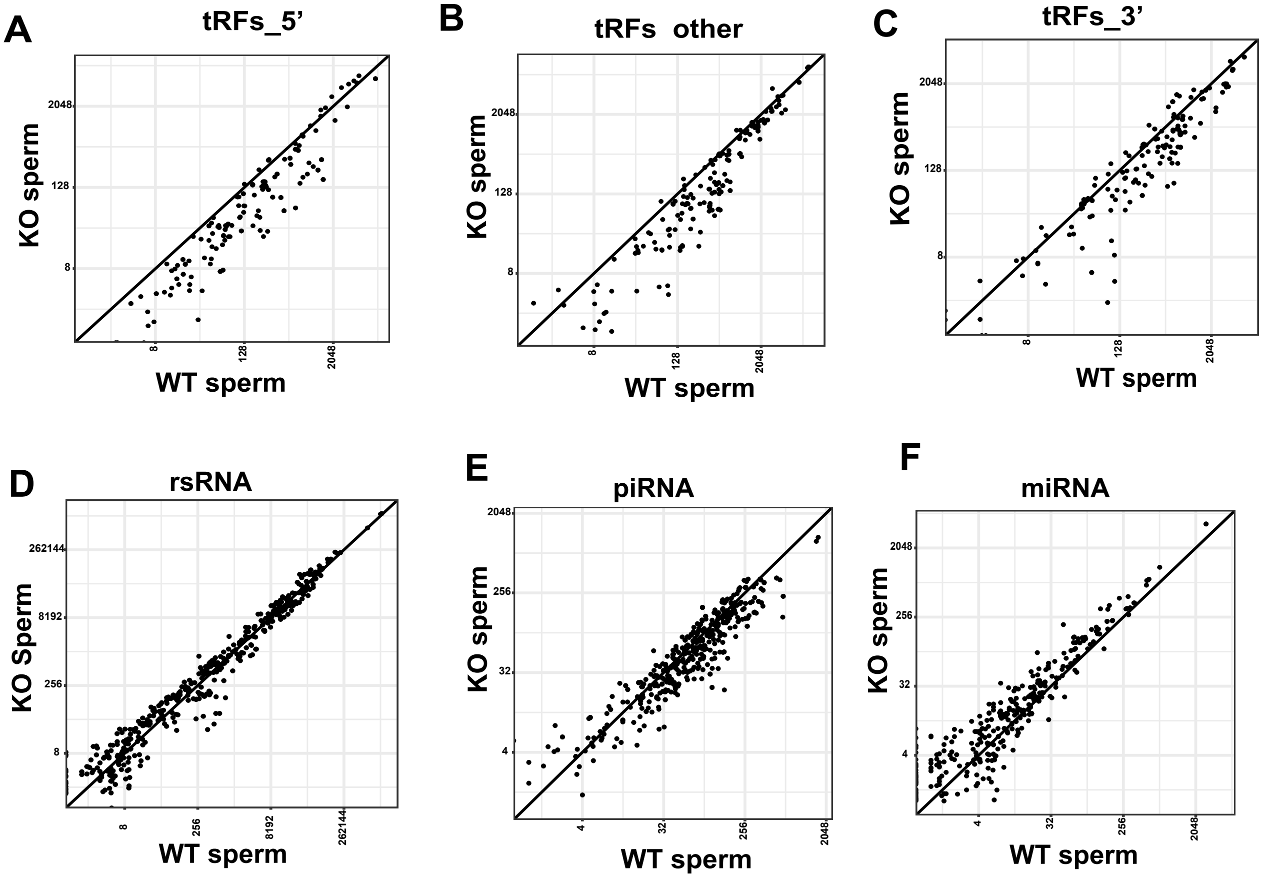

Figure S7: A) Scatter plots of transcript abundance (mean of normalized read counts) of specific small RNA classes in KO and WT sperm.
